# Advances in Sustainable γ-Valerolactone (GVL) Production via Catalytic Transfer Hydrogenation of Levulinic Acid and Its Esters

**DOI:** 10.1021/acssuschemeng.4c05812

**Published:** 2024-09-30

**Authors:** Memoona Khalid, Marta Granollers Mesa, Dave Scapens, Amin Osatiashtiani

**Affiliations:** † Energy and Bioproducts Research Institute (EBRI), College of Engineering and Physical Sciences, Aston University, Birmingham B4 7ET, United Kingdom; ‡ Luxfer MEL Technologies, Manchester M27 8LN, United Kingdom

**Keywords:** Levulinic acid, levulinate esters, catalytic transfer hydrogenation, γ-valerolactone, non-precious metals

## Abstract

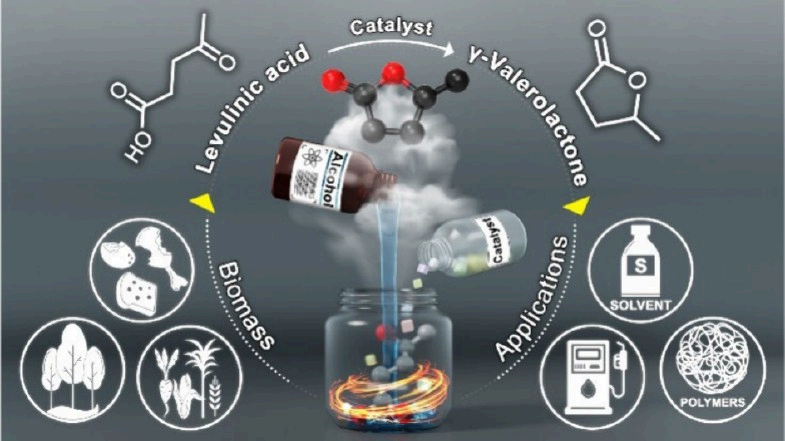

γ-Valerolactone (GVL) is a versatile chemical derived from biomass, known for its uses such as a sustainable and environmentally friendly solvent, a fuel additive, and a building block for renewable polymers and fuels. Researchers are keenly interested in the catalytic transfer hydrogenation of levulinic acid and its esters as a method to produce GVL. This approach eliminates the need for H_2_ pressure and costly metal catalysts, improving the safety, cost effectiveness and environmental sustainability of the process. Our Perspective highlights recent advancements in this field, particularly with respect to catalyst development, categorizing them according to catalyst types, including zirconia-based, zeolites, precious metals, and nonprecious metal catalysts. We discuss factors such as reaction conditions, catalyst characteristics, and hydrogen donors and outline challenges and future research directions in this popular area of research.

## Abstract 2

This article discusses catalytic transfer hydrogenation as a sustainable route for GVL production, highlighting recent advances, challenges, and future directions.

## 1 Introduction

The global dependence on non-renewable fossil reserves has exacerbated a plethora of issues relating to the scarcity of fossil resources,^1^ energy security concerns,^2^ unpredictable costs,^3^ and, most critically, the rampant and sustained degradation of the environment.^4^ These concerns, specifically those regarding climate change, have led to greater emphasis on the research and development of renewable energy carriers and chemicals. Globally, these efforts are part of broader decarbonization of energy systems programmes by 2050, with the hope of contributing to a net negative carbon future through carbon capture and bioenergy innovations.^5^ Toward these ambitious targets, biomass has a crucial role to play as a renewable carbon source with almost no net carbon emissions during its production and utilization cycle.^6^ The most abundant biomass form is lignocellulose, which is mainly composed of cellulose, hemicellulose, and lignin. These biopolymers can form the basis for the production of fuels, chemicals, and polymers, allowing for an affordable and sustainable solution to our energy and environmental concerns.

Biomass and its derivatives can be converted to more valuable products through chemical catalytic routes. Catalytic conversion of biomass-derived compounds into high-value chemicals and fuels has taken the lead in optimizing the conversion process and reducing costs, such that these products are commercially competitive with those produced from fossil reserves. This is because catalysts function to increase the rate of reaction by decreasing the activation energy of the process. However, the net enthalpy of the reaction stays the same, and the catalyst itself undergoes no permanent chemical change. Currently, extensive research has been directed toward homogeneous and heterogeneous catalysts for the catalytic transformation of biomass. Homogeneous catalysts feature active sites within the same phase as the reactants, resulting in rapid reaction rates and a high conversion rate per catalyst molecule. Nonetheless, this advantage often comes at the cost of challenging catalyst recycling and reusability. As opposed to homogeneous catalysts, heterogeneous catalysts exist in a different phase than the reactants (typically the solid phase), allowing reactions to take place at the surface, making them easier to recycle.

A useful and versatile compound that can be derived from lignocellulose is γ-valerolactone (GVL). GVL is an organic compound with a five carbon (valero)cyclic ester ring (γ-lactone). This colorless liquid possesses a sweet, herbaceous fragrance, making it valuable in applications such as perfumery and food additives. Furthermore, under normal conditions, GVL is in liquid phase and relatively stable but reactive enough to produce a variety of compounds such as butene,^7^ valeric acid,^8^ and 5-nonanone.^9^ Additionally, this stability means that GVL is resistant to decomposition or degradation with time, even in the presence of water or oxygen, without forming peroxides in the air. GVL has low toxicity (LD50 Oral-rat = 8800 mg kg^–1^) with the main risk being flammability.^10^ However, the volatility of this compound is low, which makes the flammability risk under normal conditions minimal. Table 1 presents GVL’s chemical structure and other physiochemical data.

**Table 1:**
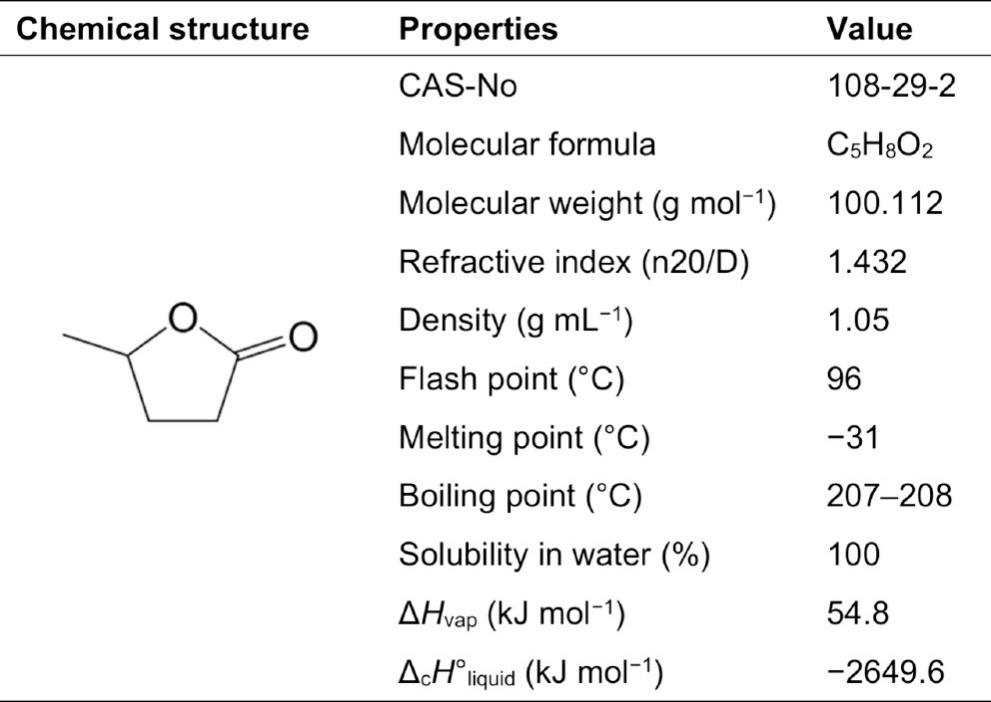
Main Properties of γ-Valerolactone (GVL)^a^

a Reproduced from ref (10) with permission from the Royal Society of Chemistry.

**Figure 1:**
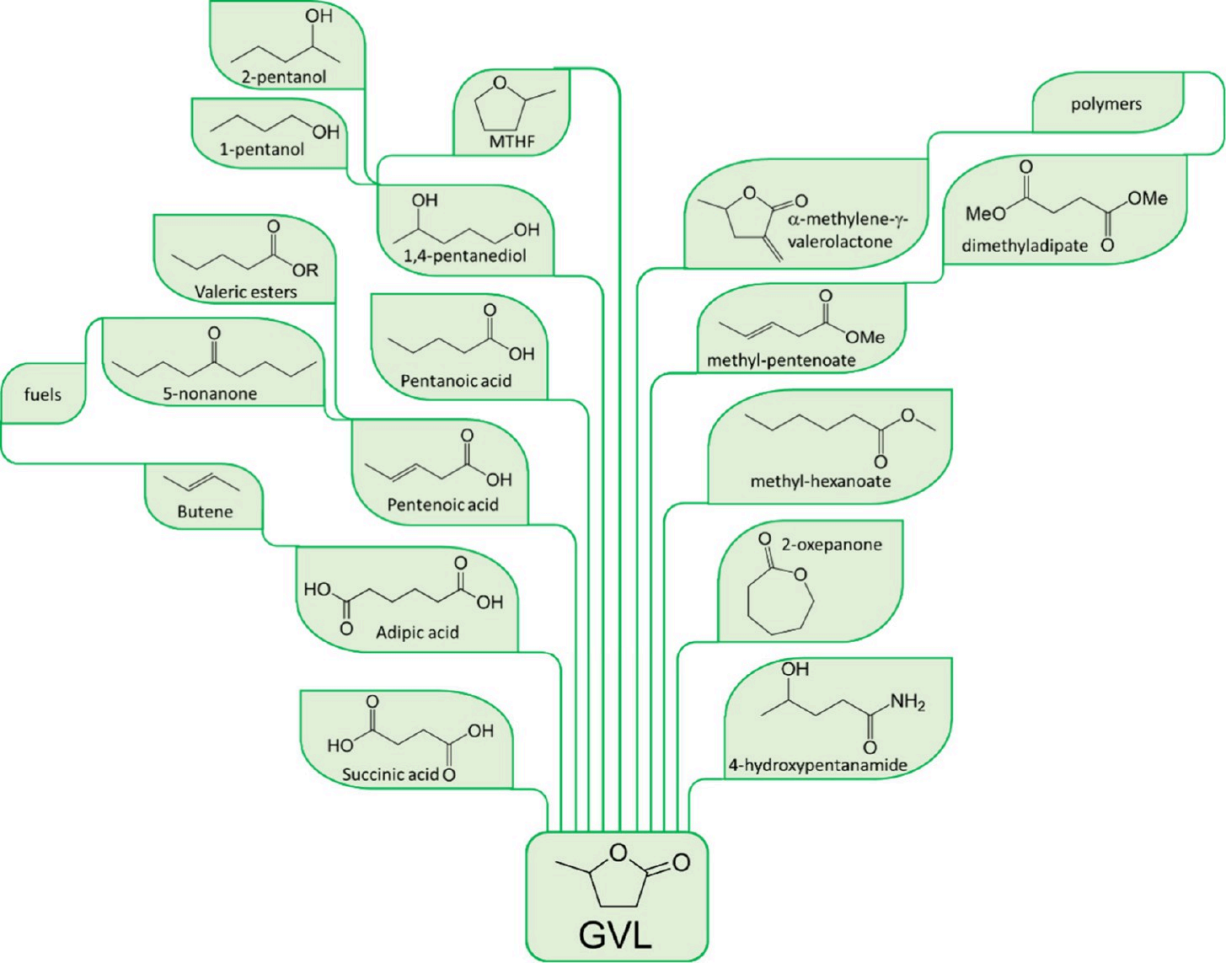
Products that could be obtained from GVL.^11,18,19^

Given its diverse range of properties, GVL can serve as an excellent and sustainable precursor for liquid hydrocarbon fuels, encompassing options such as petrol and diesel fuels, including C_8_–C_18_ alkanes and valeric esters. The synthetic pathways for these fuels are illustrated in Figure 1.^11^ GVL can also be upgraded to 2-methyltetrahydrofuran (MTHF), a flammable mobile liquid, often used as a solvent. MTHF has also shown promise as a fuel additive, with reports detailing blends of up to 70% with petrol.^12^ GVL also performs considerably well as a fuel additive, yielding results on par with those of ethanol. For example, it was reported that 10% GVL with 90% 95-octane gasoline yielded similar results to 10% ethanol blends with 90% 95-octane petrol.^13^ Among its various applications, GVL can also be employed as a green solvent in biomass processing, reducing the number of steps for biomass pre-treatment. This can be achieved through integrated catalytic conversion of hemicellulose and cellulose to furfural and levulinic acid (LA) within a single reactor.^14^ Furthermore, GVL serves as a valuable precursor for several biopolymers, including nylon,^15^ polyethers, and polyurethanes.^10^ The synthesis of nylon involves the ring opening of GVL, leading to the formation of methyl pentenoate, which subsequently serves as the building block for nylon precursors such as caprolactone, caprolactam, and adipic acid.^16^ Additionally, GVL can undergo ring opening reactions to produce γ-hydroxy(amino)amide compounds, which function as monomers in the creation of polymers like polyethers and polyurethanes.^17^

## 2 GVL Production from Lignocellulosic Biomass

Despite the growing interest in GVL as a sustainable platform chemical, its industrial production remains in its infancy. As a result, specific data on current annual global production volumes are not readily available. This limitation in data highlights the early stages of commercial development and the potential for significant growth. Yet, research into GVL production from biomass has shown significant promise, with various pathways being explored.

**Figure 2:**
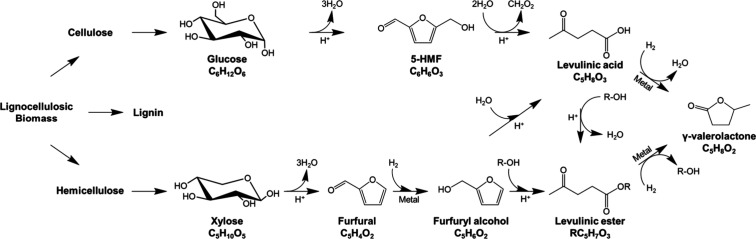
Reaction pathway for the conversion of lignocellulosic biomass to GVL. Copyright (2017) Wiley. Used with permission from ref (27).

Production of GVL from lignocellulosic biomass begins with biomass fractionation. Once the lignocellulosic biomass is fractionated into its constituents, the cellulose and hemicellulose fractions can undergo acid hydrolysis to form glucose and xylose. As illustrated in Figure 2, the C_6_ sugar, glucose, which is mainly derived from cellulose, undergoes a dehydration and rehydration process to yield formic acid^20,21^ and the desired LA, with 5-hydroxymethylfurfural (5-HMF) as an intermediate molecule. Additionally, xylose (derived from hemicellulose) can be used to produce LA via furfural and furfuryl alcohol intermediates. Following this, levulinate ester can be produced by esterification or alcoholysis of LA or furfuryl alcohol,^22−24^ respectively. Sequential hydrogenation of either of these yields the desired GVL.

In general, there are three ways to hydrogenate LA and its esters to GVL; (i) hydrogenation using molecular H_2_ gas, (ii) *in situ* hydrogen production followed by hydrogenation, and (iii) catalytic transfer hydrogenation using a hydrogen donor solvent. While each of the above routes can be catalyzed by both homogeneous and heterogeneous catalysts, the focus of this article is on the latter. We invite our readers who are interested in homogeneous catalysis for the transformation of LA to refer to a review paper by Miller et al.^25^ and a book chapter by L. T. Mika and I. T. Horváth.^26^

### 2.1 Levulinic Acid Hydrogenation Using H_2_ Gas

Platform chemicals derived from biomass tend to exhibit high oxygen content, frequently manifesting as functional groups, including alcohols, aldehydes, ketones, carboxylic acids, and similar moieties. This is due to the high presence of oxygen in the biomass. Therefore, upgrading these platform intermediates, such as LA, to value-added products and liquid fuels often requires hydrogenation processes.

**Figure 3:**
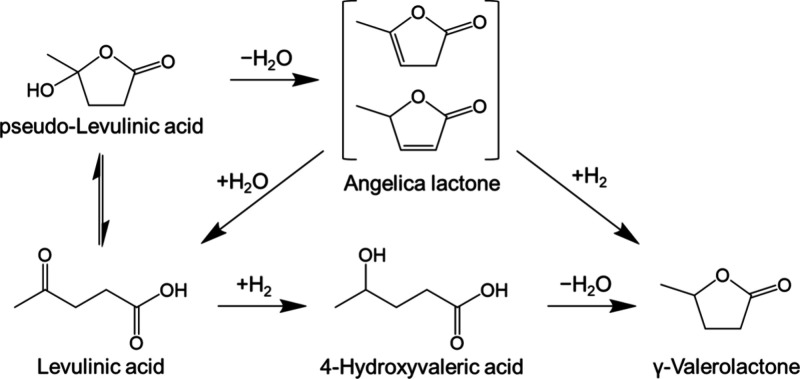
Proposed reaction pathway of LA to GVL using H_2_ gas. Copyright (2012) Wiley. Used with permission from ref (28).

Conventional hydrogenation is typically carried out using catalysts that include precious metals like Pt, Pd, and Ru or slightly more abundant metals such as Ni. There is a general consensus in the literature over how LA hydrogenation proceeds using H_2_ gas.^28−32^ It has been well-established that the cyclization of LA results in the production of pseudo-LA, which can, in turn, undergo dehydration to yield an unsaturated cyclic ester known as angelica lactone. Subsequently, an angelica lactone can be transformed into GVL through hydrogenation. The alternative proposed reaction pathway involves the direct conversion of the carbonyl group at the fourth position into a hydroxyl group, followed by a subsequent cyclization step, yielding the desired GVL (Figure 3).

However, this method of GVL production poses several challenges. First, it relies on scarce and costly metals, subject to potential supply disruptions due to geopolitical factors.^33,34^ Second, concerns have arisen regarding the toxicity of the salts and nanoparticles associated with these catalysts, especially Ni.^35^ Additionally, the process necessitates the use of high-pressure H_2_, an extremely flammable gas, as H_2_ exhibits a low solubility in most of the solvents. Therefore, achieving the desired catalyst performance often requires the application of elevated pressure and temperature levels.^36^ For instance, when hydrogenating LA, pressures exceeding 30 bar may be required, depending on factors such as the reaction temperature and catalyst type.^27^ Furthermore, for GVL production to truly uphold sustainability, it is imperative to ensure that the necessary hydrogen is derived from renewable and environmentally friendly sources. According to the International Renewable Energy Agency, as of the conclusion of 2021, global hydrogen production figures stand at approximately 47% from natural gas, 27% from coal, 22% from oil (often as a byproduct), with a relatively modest contribution of around 4% originating from electrolysis.^37^ These concerns regarding the economic feasibility, safety, and sustainability of commercial GVL led to intensified research activities on alternative methods for GVL production.

### 2.2 *In Situ* Hydrogenation of Levulinic Acid Using Formic Acid

A different approach for GVL production from LA entails the utilization of formic acid as a hydrogen source, in contrast to the conventional use of H_2_ gas. Formic acid can serve as a hydrogen source in an *in situ* process. In this process, formic acid is decomposed into CO_2_ and H_2_, and the reactively formed H_2_ is then employed to convert LA into GVL. In the process of producing LA from lignocellulosic biomass, one mole of formic acid is generated as a byproduct for every mole of LA produced.^14,38^ Utilizing this byproduct as the hydrogen source in the subsequent hydrogenation step to convert LA into GVL eliminates the necessity of a LA purification step. As specific cost reductions depend on various factors, such as the type of feedstock, catalyst, and operating conditions, it is difficult to quantify the specific cost reductions without detailed economic analysis. However, this process simplification is expected to contribute to a reduction in the overall cost of GVL production. Additionally, the use of bio-based formic acid as the hydrogen source obviates the requirement for hydrogen gas, which may otherwise be obtained from non-renewable sources.

On the other hand, it must be noted that the decomposition of formic acid occurs through two competitive pathways: dehydrogenation (Reaction R1) and dehydration (Reaction R2).^39^
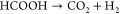
R1
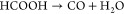
R2

The presence of CO in the reaction medium can further promote additional side reactions.^40^ Therefore, to ensure optimal efficiency in the process, it is crucial to achieve the selective conversion of formic acid into H_2_ and CO_2_. This requires the development of catalysts that promote dehydrogenation while suppressing the dehydration of formic acid. Furthermore, to obtain reasonable GVL yields, it is often necessary to use formic acid in excess amounts,^41^ which will in turn adds to the overall cost of the process and raises concerns about the efficient utilization of resources. The use of excess formic acid also complicates the downstream processing as the unreacted formic acid must be separated and ideally recycled. The presence of formic acid can also promote the leaching of active sites from the catalyst, leading to catalyst deactivation.^42,43^ While *in situ* hydrogen production via formic acid decomposition may reduce the number of processing steps leading to lower operation costs, this process still requires the use of precious metal catalysts, or harsh reaction conditions (*T* > 200 °C). Additionally, if the goal is to reduce CO_2_ emissions, then this process might not be desirable. The reactively-formed CO_2_ during the decomposition of formic acid is biogenic and thus can be considered carbon-neutral. However, in order to combat climate change, negative carbon emissions are becoming increasingly crucial, because they will help withdrawing CO_2_ from the atmosphere, thereby undoing the cumulative effect of historical emissions. This would require the process to be coupled to carbon capture and storage (CCS) technologies. Apart from increasing the cost, CCS technology increases the complexity of the process, hence, making it more challenging in practice. Hijazi et al. and Yu et al. have conducted comprehensive literature reviews on the hydrogenation of LA using formic acid as the hydrogen source.^39,41^ For readers seeking in-depth information on this subject, we recommend that readers refer to these review articles.

### 2.3 Catalytic Transfer Hydrogenation of Levulinic Acid and Its Esters

**Figure 4:**
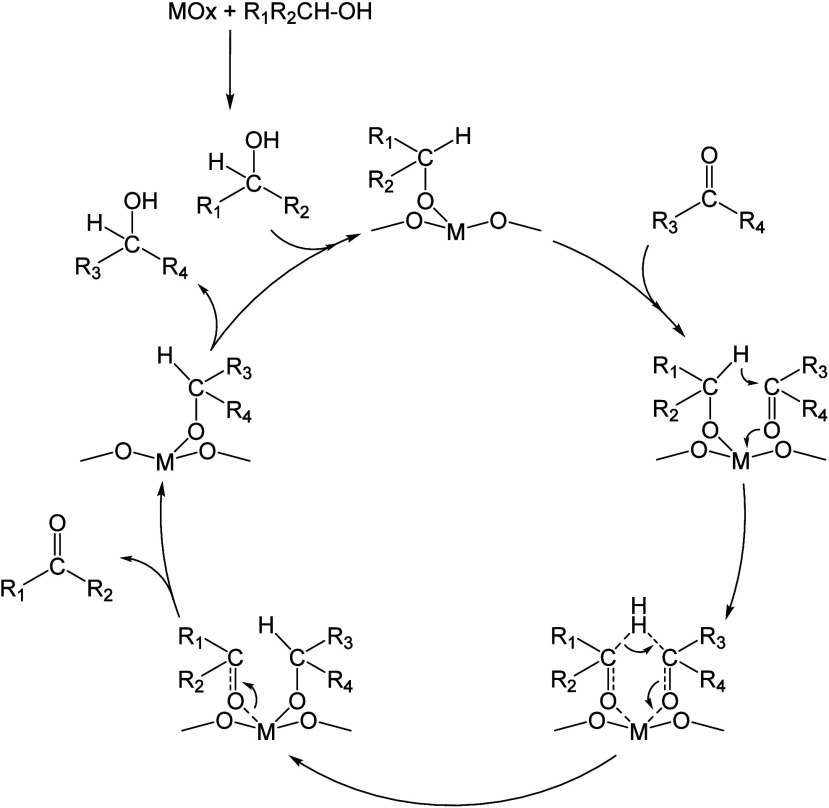
Mechanism of metal oxide catalyzed MPV reduction of ketones and aldehydes.^45−47^

The pursuit of more sustainable and cost-effective LA hydrogenation methods has led to the exploration of the cooperative use of heterogeneous catalysts and liquid renewable H_2_ sources (e.g., renewable alcohols) via a process known as catalytic transfer hydrogenation (CTH). This method follows the Meerwein–Ponndorf–Verley (MPV) reaction, which involves the reduction of carbonyl group to a corresponding hydroxyl group through the transfer hydrogenation from an alcohol.^44^ This process negates the use of expensive metal catalysts and a molecular H_2_. The reaction mechanism (Figure 4) involves the transfer of α-H to the α-C of the alcohol and simultaneous transfer onto the carbonyl carbon in a concerted reaction that proceeds via a six-membered ring intermediate.^45^

**Figure 5:**
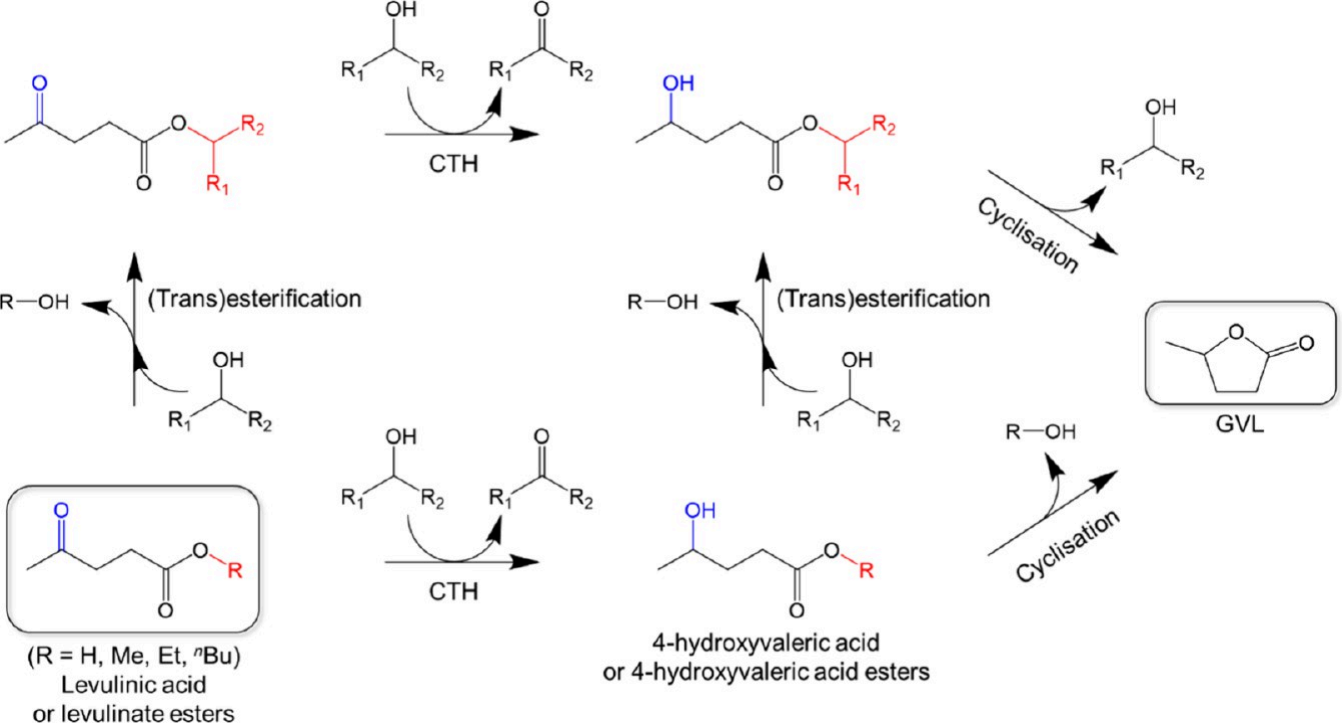
Most widely accepted reaction pathways for the catalytic transfer hydrogenation (CTH) of levulinic acid and its esters using secondary alcohols as H-donors.^48−51^

This reaction is advantageous for the high chemoselectivity in reducing carbonyl groups despite the presence of other functional groups (i.e., C=C bonds) even under mild conditions. It has been suggested that the conversion of LA and its esters to GVL using secondary alcohols, such as 2-propanol, can occur through two pathways. As illustrated in Figure 5, one pathway involves 4-hydroxyvaleric acid or its ester derivative, depending on the starting material, via the mechanism mentioned earlier. Subsequently, cyclization of the intermediate compound leads to the production of GVL. The alternative route involves the esterification of LA (or transesterification of levulinate ester) into the corresponding iso-alkyl levulinate ester. This intermediate product then undergoes CTH reaction and cyclization to yield GVL.^48−51^

**Figure 6:**
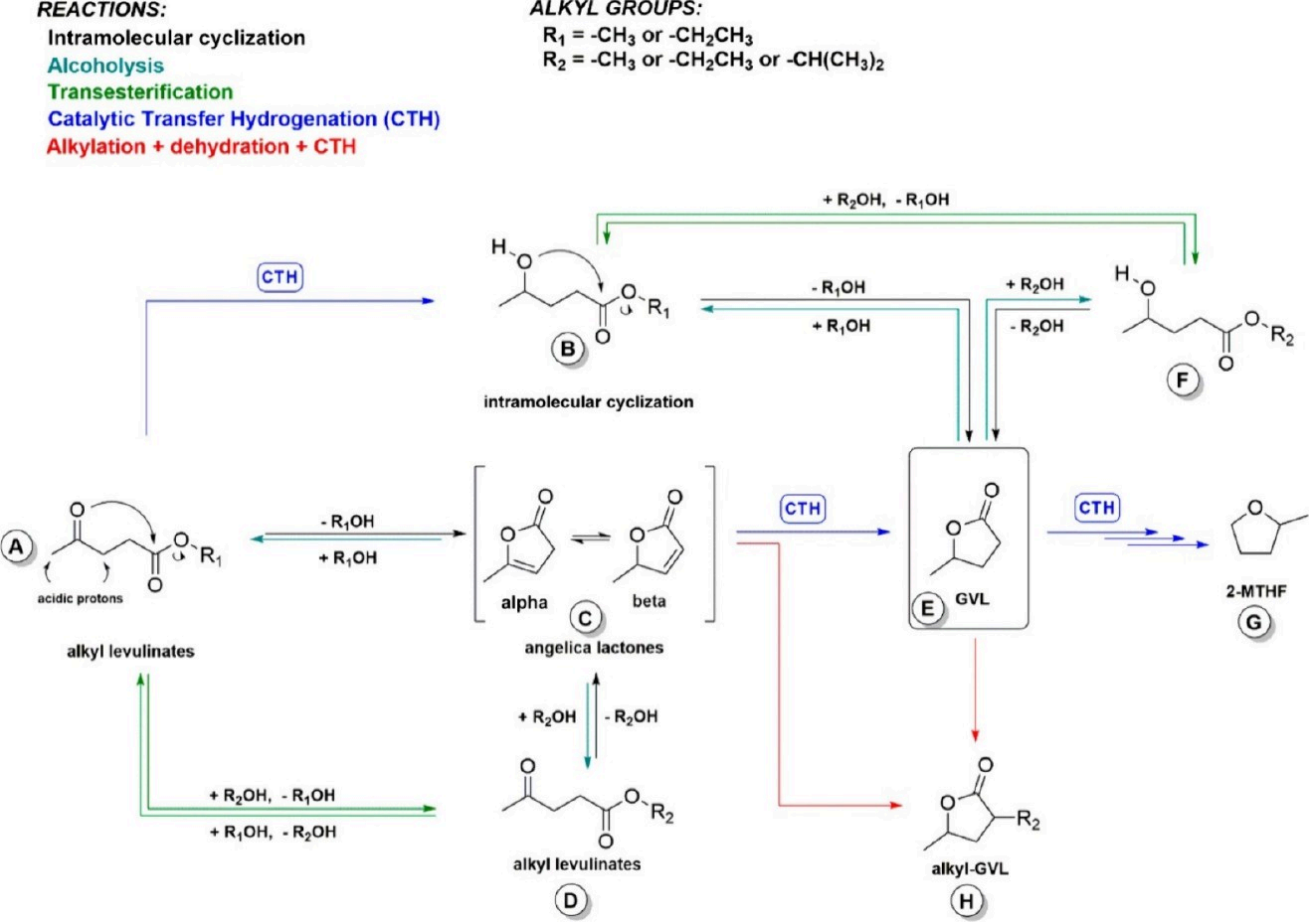
Reaction pathways for CTH of alkyl levulinates proposed by Tabanelli et al. Reproduced with permission from ref (52). Copyright (2019) American Chemical Society.

These are the two widely accepted reaction pathways typically observed in liquid phase CTH reactions; however, Tabanelli et al.^52,53^ were able to identify more intermediates and products when the reaction of levulinate esters were performed in the gas phase at temperatures >200 °C. This enabled them to propose an additional reaction pathway, as illustrated in Figure 6. The new proposed route proceeds via CTH of α- and β-angelica lactones (the latter being formed by the cyclization of alkyl levulinates), which, in turn, may undergo alcoholysis leading to alkyl levulinate (e.g., isopropyl levulinate). Formation of alkyl levulinate (Product D in Figure 6) and GVL from angelica lactones was confirmed by allowing the β-angelica lactone to react with different alcoholic solvents. It is worth noting the possibility of other side reactions that can take place when using sacrificial hydrogen donor solvents. For example, López-Aguado et al. reported the formation of diisopropyl ether when using 2-propanol as the hydrogen donor in the presence of a bifunctional Zr–Al–Beta catalyst.^54^

In this Perspective article, we categorize the various catalysts explored for GVL synthesis via CTH reaction into four main groups: zirconia-based, zeolites, precious metals, and non-precious metal catalysts. Our focus is on the most recent advancements in this field, specifically covering research published after 2017. For earlier studies, we direct readers to our previous review published in 2017.^27^

#### 2.3.1 Zirconia-Based Catalysts

Zirconia-based catalysts have shown significant promise in the CTH of LA and its esters to GVL. Their relatively low cost makes them particularly attractive for industrial and commercial applications. For these reasons, many researchers have focused on these catalysts, experimenting with different formulations and reaction conditions to improve efficiency and better understand their structure–activity relationship.

**Figure 7:**
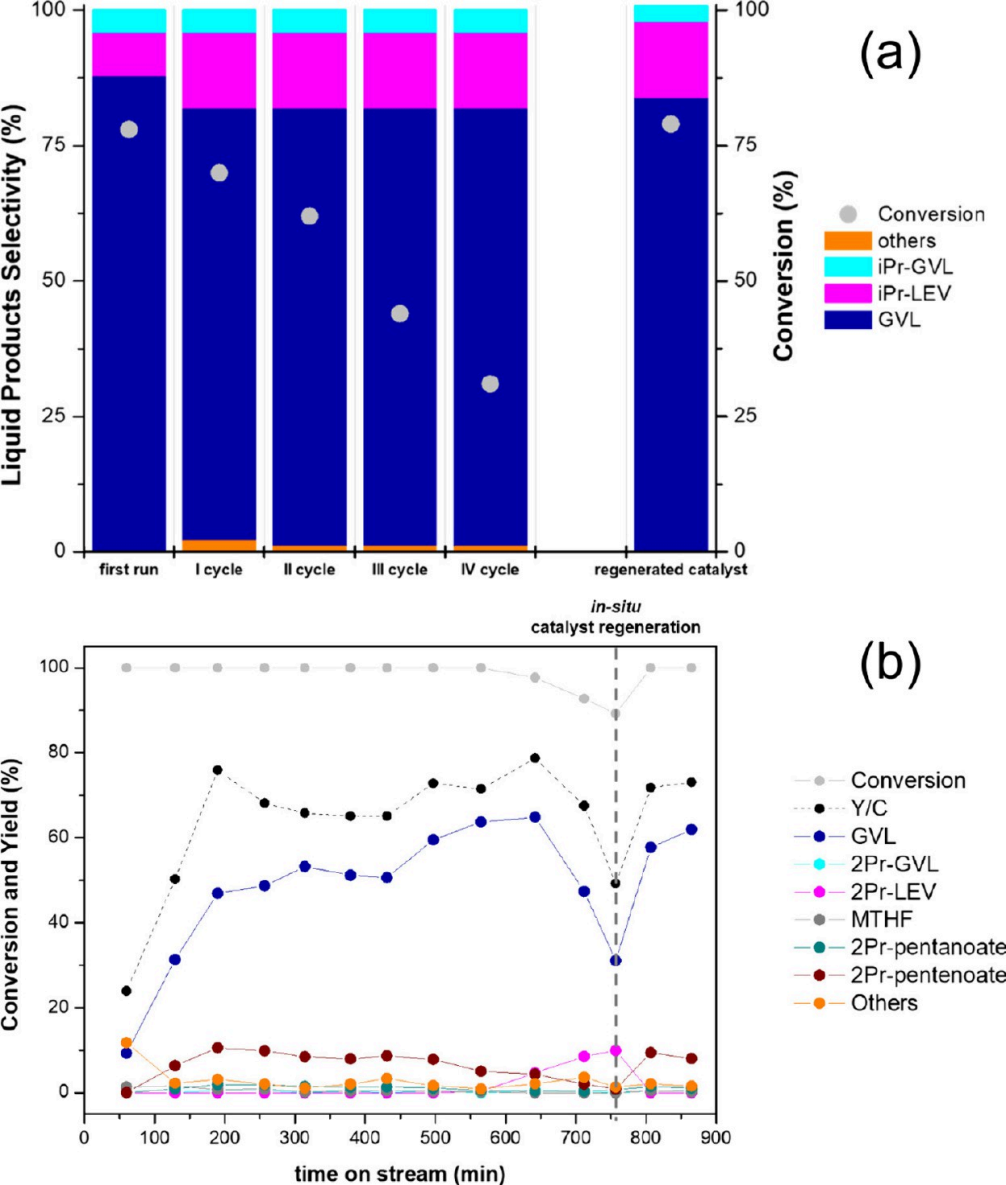
Products distribution and stability of ZrO_2_ catalyst in CTH of EL using 2-propanol as a H donor. a) In liquid phase in batch mode (reaction conditions: 0.30 g ZrO_2_, 40 mL solution of ethyl levulinate (10 wt %), 250 °C, 8 h, N_2_ pressure 10 bar, stirring 500 rpm). b) In continuous gas phase (reaction conditions: molar ratio EL:2-propanol = 1:10, 200 °C, τ = 1 s, mol % N_2_:EL:alcohol = 90.1:0.9:9). Reproduced with permission from ref (52). Copyright (2019) American Chemical Society.

Tabanelli et al. performed a comparison of batch versus continuous flow gas phase CTH of methyl levulinate (ML) and ethyl levulinate (EL) using methanol, ethanol, or 2-propanol as the hydrogen donor, and a ZrO_2_ catalyst prepared by precipitation of a zirconium(IV) nitrate dihydrate precursor.^52^ Under batch conditions of 250 °C, 10 bar N_2_ pressure, and 0.3 g of catalyst, EL conversion was generally higher than that for ML, and 2-propanol was identified as a better H donor compared to ethanol and methanol. Using 2-propanol as the H donor, they achieved 31% EL conversion with 27% GVL yield (87% GVL selectivity) under these conditions. Exploring the catalytic performance of ZrO_2_ under continuous gas-flow conditions demonstrated high conversions and GVL yield using 2-propanol and ethanol but rather a poor reactivity with methanol. Regarding the catalyst stability, a considerable decline in catalyst activity was observed in the batch mode when it was recycled (Figure 7a); however, the activity could be regained by recalcining the catalyst, suggesting the deposition of carbonaceous species on active sites as the main deactivation mechanism. When operating in the continuous mode (Figure 7b), the catalyst was stable for up to 10 h, after which it was regenerated *in situ* by feeding air at 400 °C.

With an aim to unravel the structure–activity relationships of zirconia monoclinic (*m*) and tetragonal (*t*) phases in the CTH of ML with ethanol, Bacchiocchi et al. focused on the gas phase reaction in a continuous flow reactor and complemented their work with density ^1^H NMR relaxation studies and functional theory (DFT) modeling.^55^ Performing the reaction at 250 °C, complete ML conversion was achieved after 120 min on stream over both *m*-ZrO_2_ and *t*-ZrO_2_, with GVL yields of 63% and 66%, respectively. After 200 min on stream, both catalysts began to exhibit a loss of activity, which the authors attributed to the deposition of heavy carbonaceous residues on the catalysts’ surfaces. However, this decline was significantly more pronounced in the case of *m*-ZrO_2_. Furthermore, *ex-situ* low-field NMR relaxation measurements were performed to assess how the interaction of species present during the reaction with the different phases of ZrO_2_ impacts the catalytic performance. The ratio of the spin–lattice to spin–spin relaxation time (*T*_*1*_*/T*_*2*_) was used as an indicator of the affinity of different molecules of interest to the catalysts. Among the tested molecules (methanol, ethanol, ML, EL, α-angelica lactone, and GVL), ethanol and methanol exhibited the strongest interactions with ZrO_2_. Between the two phases of zirconia, *t*-ZrO_2_ adsorbed both alcohols more strongly than did *m*-ZrO_2_. Considering the catalytic testing results that demonstrated a higher GVL yield and catalyst stability for *t*-ZrO_2_, the authors concluded that a stronger interaction of the alcohols with ZrO_2_ “activates” the alcohol molecules, enhancing their H-donating ability and thus boosting catalytic activity and stability. By increasing the molar excess of ethanol to ML to 20, both phases demonstrated improved stability, with *t*-ZrO_2_ showing almost negligible deactivation. Additionally, angelica lactone was identified as the main precursor for the deposition of carbonaceous species on the catalysts. Combined with temperature-programmed desorption (TPD) and DFT studies, it was concluded that the faster deactivation of *m*-ZrO_2_ is a consequence of higher Lewis acidity and basicity, which leads to stronger interactions with the intermediate angelica lactone.

**Figure 8:**
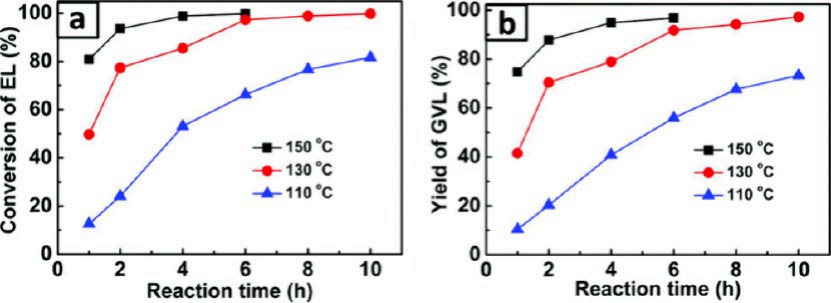
Influence of reaction temperature on a) EL conversion and b) GVL yield using porous zirconium–cyanuric acid polymer as a catalyst. Reaction conditions: liquid phase reaction in batch mode using 0.2 g Zr–CA, 1 mmol EL, 6 g of 2-propanol. Reproduced from ref (56) with permission from the Royal Society of Chemistry.

Xue and co-workers recognized that the basicity of the catalyst has a resounding influence on the progress of reaction and so they set about synthesizing porous zirconium–cyanuric acid polymer (Zr–CA) with basic groups and metal–ligand coordination possibilities.^56^ When compared to the ZrO_2_ catalyst, the higher Lewis acidity and basicity afforded by cyanuric acid led to higher activity and selectivity. An EL conversion of 89% and a GVL yield of 82% was achieved after reacting at 130 °C for 4 h (Figure 8) with a recyclability of up to five cycles. Comparing the reactivity of EL and LA, both reactants reached full conversion with a similar 97% GVL yield at 130 °C. Interestingly, LA achieved this within 4 h of reaction time, while EL required 10 h. The rapid reaction rate of LA was attributed to its self-acidity, which could enhance the lactonization of 4-hydroxyvaleric acid intermediate (Figure 5).

**Figure 9:**
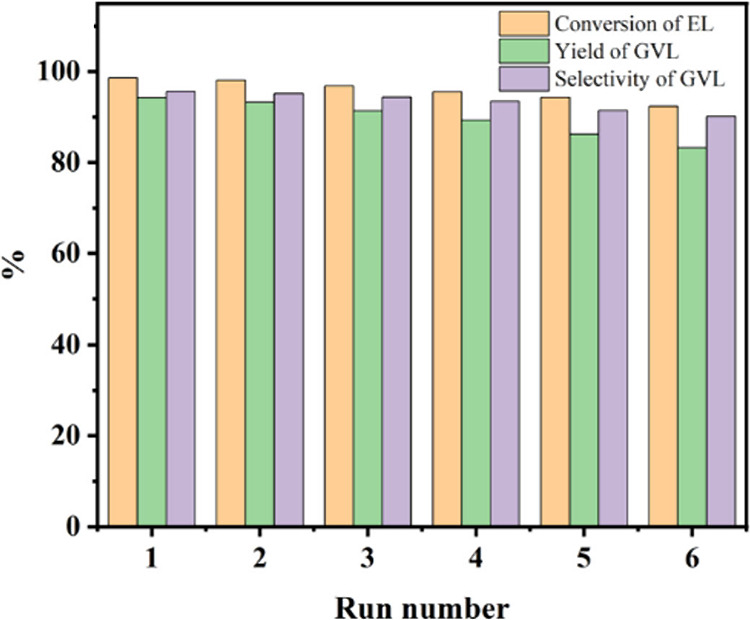
Recycle experiment of Zr-GA. Reaction conditions: liquid phase reaction in batch mode using 0.144 g EL (1 mmol), 0.2 g of catalyst, 5 mL of 2-propanol, 160 °C, 8 h.^57^ Reproduced with permission from Springer Nature.

Other organic–inorganic bifunctional catalysts have been developed, including a Zr-based catalyst with gallic acid (Zr–GA) by Li and colleagues.^57^ The catalyst was synthesized via coprecipitation of ZrCl_4_ and gallic acid upon which catalysis proceeded at 160 °C for 8 h in the presence of 2-propanol for a 99% EL conversion and 94% GVL yield. Li et al. was able to capitalize on the synergistic effect from Zr^4+^ Lewis acid sites, phenolic hydroxyl Brønsted acid sites, and phenolate Lewis base sites to reach high activity and selectivity. Reusability of up to six cycles was studied without a significant decrease in activity or selectivity (Figure 9).

In a report completed by Li et al., transfer hydrogenation of LA and its esters was conducted using zirconium phosphate (Zr-PO) catalysts with varying ratios of Lewis and Brønsted acid sites.^58^ Experiments proceeded at 210 °C for 2 h with butyl levulinate (BL) as the substrate and 2-propanol as the hydrogen donor. With a Lewis/Brønsted ratio of 4.7, the catalyst ZrPO-1.00 outperformed with 98% conversion and 96% GVL yield. A decrease in Lewis/Brønsted acid sites ratio led to a large decrease in BL conversion and GVL selectivity, demonstrating that in CTH reactions, catalysts must have sufficient Lewis acidity for optimum conversion and yield.

Similarly, Xie et al. also investigated the potential of phosphate groups in CTH by synthesizing zirconium trimetaphosphate (Zr-TMPA).^59^ Zr-TMPA was produced using sodium trimetaphosphate and ZrOCl_2_ and was used during 8 h GVL synthesis reaction at 160 °C with 2-propanol as the hydrogen donor solvent. Zr-TMPA showed the highest activity with full conversion of EL and a GVL yield of 96%. The O^2–^ basic sites from the phosphate groups combined with Zr^4+^ acid sites contributed to the high activity and selectivity observed.

**Figure 10:**
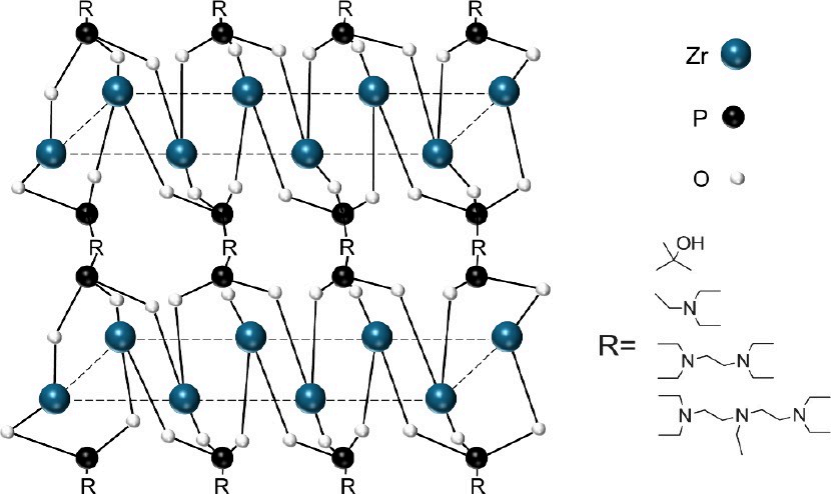
Plausible structures of metal phosphonate materials. Copyright (2018) Wiley. Used with permission from ref (60).

Organic zirconium phosphonate materials have also been a subject of interest for researchers.^60^ Wang et al. synthesized a range of metal phosphonate catalysts (Figure 10) using organic phosphonic acid sodium salt and ZrOCl_2_ in water to be used in 12 h reactions at 160 °C with EL substrate and 2-propanol solvent. The zirconium hydroxyethylidene diphosphonic acid (Zr-HEDP) catalyst outperformed the others with 99% conversion and 92% GVL yield. The Zr-HEDP catalyst contained more Brønsted acid sites stemming from the hydroxyl groups on the surface, which along with the Lewis acidity afforded by the Zr were crucial to EL conversion. Furthermore, the enhanced basicity from the N atoms of the phosphonate groups allowed for greater catalytic activity.

**Figure 11:**
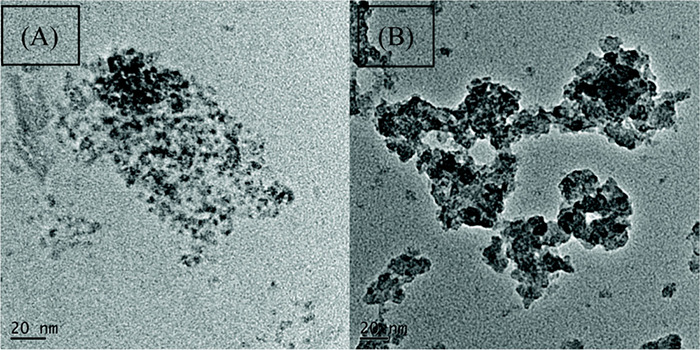
TEM images of (A) GluPC and (B) GluPC-Zr. Reproduced from ref (61) with permission from the Royal Society of Chemistry.

In another more recent study, Wan et al. synthesized glucose phosphate carbamide zirconium (GluPC-Zr) catalysts using a two-step method.^61^ The first step involved the synthesis of glucose phosphate carbamide (GluPC) by mixing phosphoric acid and urea. Then, upon precipitation with methanol, GluPC was collected and reacted with ZrCl_4_. Transmission electron microscopy (TEM) images are shown in Figure 11, which demonstrate the high porosity of the GluPC-Zr catalyst upon reaction of the GluPC precursor with ZrCl_4_. This catalyst also exhibited an enhanced Lewis acid–base properties which resulted in the complete conversion of LA to 95% GVL yield in a 12 h reaction at 180 °C with 2-propanol. It was observed that with a further increase in the reaction temperature (190 °C) a GVL yield of 98% was achieved.

Widespread interest has been directed to the performance of silica supported ZrO_2_ for the CTH of LA and its esters. Kuwahara’s group tested a series of SBA-15-based supported catalysts with 10 to 60 wt % ZrO_2_ loadings for a CTH reaction with ML.^48^ It was found that the 10 wt % ZrO_2_ loading had enhanced ML conversion and GVL yield. The better performance of the low loading catalyst was attributed to higher dispersity of Zr atoms on silica. Specifically, the highly dispersed Zr^4+^ oxide compounds with lower coordination capacities were the principal active species. Reusability tests revealed ZrO_2_/SBA-15 catalysts were more stable compared to bulk ZrO_2_ due to the high Zr dispersity stabilized over large SBA-15 surface area, which prevented over accumulation of organic matter and allowed for easy access to active sites.^48^ In works by Enumula and co-workers, SBA-15 loaded with 10–30 wt % ZrO_2_ catalysts were synthesized by a wet impregnation method for transfer hydrogenation of LA in 2-propanol 250 °C and 1 atm N_2_ pressure.^62^ Despite achieving full LA conversion exclusively with SBA-15, it should be noted that GVL selectivity was greatly improved with increased ZrO_2_ loading, reaching 93% over a 23% ZrO_2_/SBA-15 sample. In addition, stability tests were conducted at longer reaction times, which revealed a 10% reduction in GVL selectivity over 20 h. However, once samples were calcined in air to remove carbonaceous deposits, the catalyst performance recovered. Analysis of the spent catalyst revealed an overall decrease in acidity resulting from the poisoning of weak and moderate acidic sites by carbonaceous deposits.

**Figure 12:**
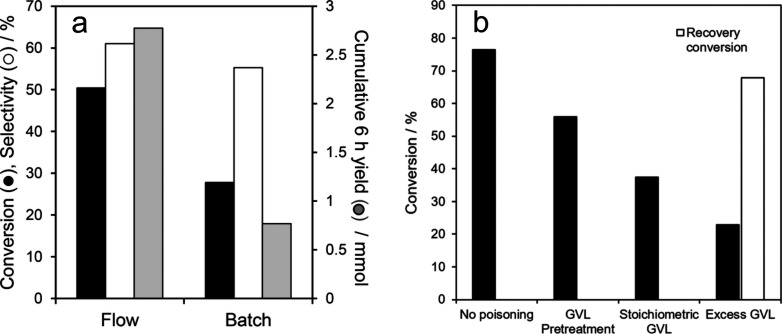
a) Comparison of liquid phase flow and batch EL transformation to GVL catalyzed by 11.6 wt % Zr/SBA-15 and b) comparison of EL conversion after 2 h reaction over 11.6 wt % Zr/SBA-15 in the absence of GVL and after: 30 min pretreatment in pure GVL at 150 °C; addition of stoichiometric GVL; addition of excess (5:1) GVL; or addition of excess GVL and subsequent removal. Reproduced from ref (49) with permission from the Royal Society of Chemistry.

In a more recent study by Osatiashtiani and co-workers, the transfer hydrogenation of EL proceeded in batch and continuous flow over ZrO_2_/SBA-15 catalysts, where conformal ZrO_2_ adlayers were dispersed on mesoporous SBA-15 silica supports.^49^ Tuning the Lewis and Brønsted acid sites (11.6 wt % Zr) afforded better EL conversion and GVL selectivity for optimization of the two-step cascade reaction. At lower ZrO_2_ surface coverages, Lewis acidity dominated over Brønsted acidity, which only emerged on completion of a ZrO_2_ monolayer. From surface characterization of catalysts, it was determined that cooperativity between Lewis acid–base pairs and Brønsted sites in the ZrO_2_ monolayer bore significance for the activity and GVL selectivity, which reached 70%, equivalent to a 40% GVL yield at 170 °C. When comparing continuous flow versus batch operation (Figure 12a), continuous flow displayed higher EL conversion and higher GVL selectivity. This is because operating in flow reduced the reversible deactivation of the Lewis acid sites by the GVL product. Lewis acid sites are essential for the catalytic transfer hydrogenation of EL to the hydroxyvalerate intermediate. As a result, the reaction in flow was not controlled by the CHT step, but by the dealcoholization and cyclization of the hydroxyvalerate intermediate to GVL, which is Brønsted acid-catalyzed (Figure 12b). Furthermore, in continuous flow GVL productivity as well as turnover frequency (TOF) significantly improved with a productivity of 5.2 mmol g^–1^ h^–1^ and a TOF of 14.5 h^–1^ being achieved compared to batch, which had a productivity of 1.37 mmol g^–1^ h^–1^ and a TOF of 3.6 h^–1^. This study demonstrated the effectiveness of flow chemistry for the accelerated production of valuable bioderived molecules.

**Figure 13:**
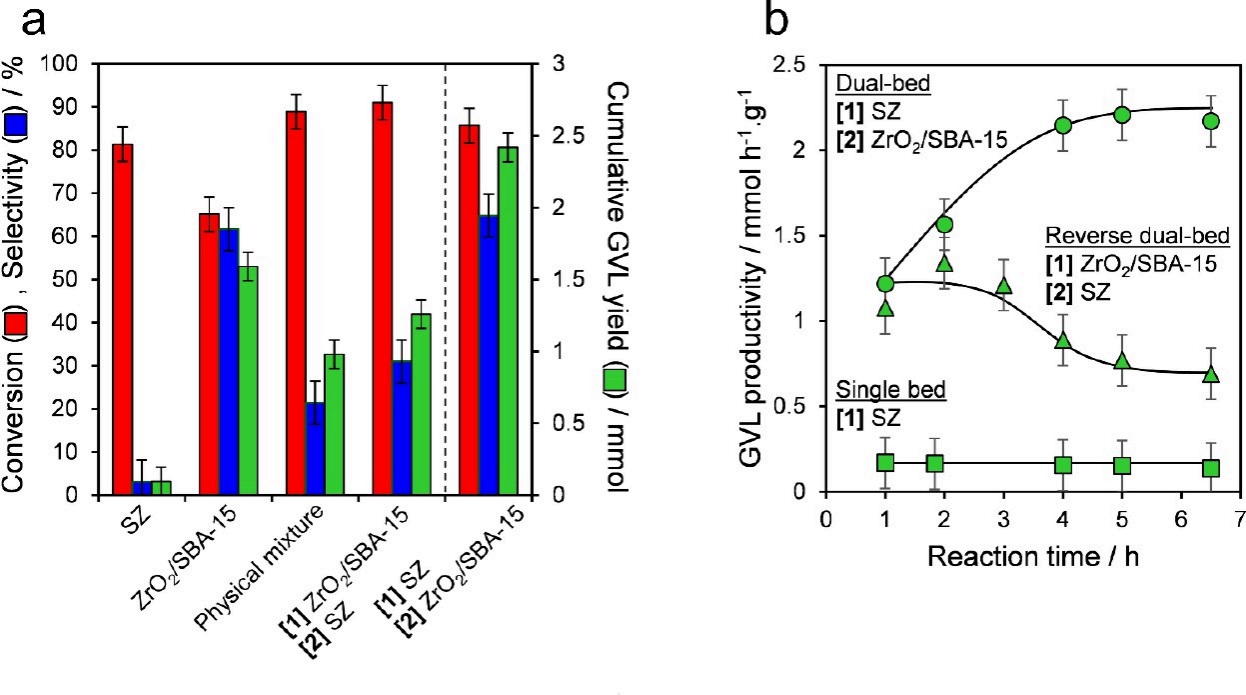
Cascade conversion of LA to GVL. a) Liquid phase continuous flow over single bed SZ catalyst with 2.6 wt % sulfur surface content, single bed commercial ZrO_2_/SBA-15 catalyst, sequential dual-bed of SZ+SBA-15, and batch conversion over physical mixture of SZ catalyst with 2.6 wt % sulfur surface content+Zr/SBA-15. b) Comparison of liquid phase continuous flow productivity for the conversion of LA to GVL employing SZ catalyst with 2.6 wt % sulfur surface content (single bed) or SZ catalyst with 2.6 wt % sulfur surface content+Zr/SBA-15 catalyst over dual-bed configuration. Liquid phase continuous flow reaction conditions: τ = 50 min total for single and dual beds, 150 °C; 100 mg of catalyst (single bed), 200 mg of catalyst (dual-bed); 2-propanol solvent. Batch reactions employing a physical mixture of SZ catalyst with 2.6 wt % sulfur surface content+Zr/SBA-15 were conducted under the same conditions, with a total duration of 6.5 h. Copyright (2023) Wiley. Used with permission from ref (50).

In later works by Merenda et al., a dual-catalyst bed configuration was examined for the liquid phase, continuous flow esterification, and successive CTH of LA to GVL.^50^ Here, a catalyst was synthesized with enhanced synergy between Brønsted acid sites in sulfated zirconia (SZ) and Lewis acid sites in ZrO_2_/SBA-15. The high sulfate saturation with dense packing of strong Brønsted acid sites allowed for optimal LA esterification to isopropyl levulinate. The ZrO_2_ bilayer, deposited over a SBA-15 mesoporous silica afforded better Brønsted/Lewis acidity for the conversion of levulinate ester and subsequent dealcoholisation/cyclization to GVL (56% yield). Using a dual bed system at 150 °C a maximum stable productivity of 2.2 mmol_GVL_ g_cat_^–1^ h^–1^ was observed. This showed significant improvements in performance for either the catalyst alone or as a physical mixture of both (Figure 13).

It has been established that the combined Lewis acidity and basicity of a catalyst has a notable effect on the conversion of LA and its esters to GVL. *In lieu* of these reports, Li et al. sought to assess the catalytic ability of basic zirconium carbonate in comparison with other basic metal carbonates such as Ni, Mg, Zn, and Pb.^63^ Experiments were conducted at 180 °C for 3 h with 2-propanol as the H donor and solvent and EL as the substrate. As theorized, the zirconium carbonate did exhibit the greatest catalytic activity, giving complete EL conversion and 96% GVL yield. This again was attributed to the cooperative effects of Lewis acid sites (Zr^4+^) and basic sites (carbonates and hydroxides). Additionally, this catalyst displayed high recyclability of up to six consecutive cycles while still maintaining good activity and selectivity.

**Figure 14:**
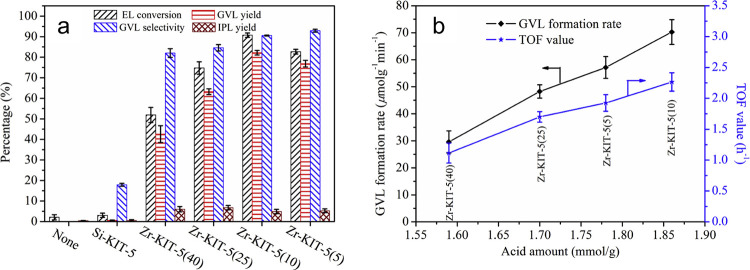
a) Activity of Zr/KIT-5 catalysts in the CTH of EL to GVL using 2-propanol as the hydrogen donor solvent. Reaction conditions: liquid phase reaction in batch mode using 1 mmol EL, 0.1 g catalyst, 5 mL 2-propanol, 180 °C, 4 h. b) GVL formation rate and TOF value as a function of acid amount where both formation rate and TOF value were calculated from the GVL yield obtained after 1 h, wherein formation rate was defined as (mole of GVL)/(gram of catalyst × time) and TOF value was calculated as (mole of GVL)/(mole of acid sites × time). Reproduced from ref (64). Copyright (2020), with permission from Elsevier.

Another example of silica-supported zirconia catalysts for CTH of biomass-derived molecules to GVL is the work by He et al. in which they utilized mesoporous silica KIT-5 as the support.^64^ They demonstrated that by tuning the Zr incorporated into KIT-5, they could control the Lewis/Brønsted acid character of the catalyst. They observed that a Si:Zr molar ratio of 10 provided the optimum balance between the two types of acid sites, exhibiting 94% EL conversion with 85.5% GVL yield at 180 °C after 6 h (Figure 14). Besides maintaining the suitable balance between Lewis and Brønsted acidity, the catalyst’s effective performance was credited to its significant quantity of acid sites (1.86 mmol g^–1^) and surface area (646.3 m^2^ g^–1^). This effectiveness was further facilitated by the readily accessible active sites, which stem from the intrinsic cage-type pores within the KIT-5 material. These pores contributed to the appropriate pore volume and microporosity and/or adsorption effect for ketone carbonyl of EL.

In another study, He et al. prepared and tested a series of Al_2_O_3_–ZrO_2_ catalysts for EL conversion to GVL.^65^ Al–Zr mixed oxide catalysts were prepared by a coprecipitation of zirconium and aluminum with varying Al/Zr molar ratios. Other reaction parameters were tested (such as reaction temperatures, reaction times, and different solvents) as well as catalyst composition relating to catalyst loading and calcination temperatures. Optimal catalytic performance was observed with the Al_7_Zr_3_ catalyst, which was calcined at 300 °C and reacted at 220 °C for 4 h with 2-propanol as a hydrogen donor. A 96% EL conversion and 83% GVL yield were reported under these reaction parameters. The enhanced catalytic activity was attributed to larger surface areas and an increase in acid and base sites compared to ZrO_2_ catalyst.

A Zr-TiO_2_ catalyst, synthesized by Zhao et al., was also effective in the conversion of EL to GVL using 2-propanol as the H donor.^66^ The 10 wt % Zr-TiO_2_ catalyst, with an average 4–6 nm nanoparticle size, was produced using a facile sol–gel hydrothermal method and utilized at 190 °C for 5 h, resulting in 79% EL conversion and 74% GVL yield. High catalytic activity was attributed to the optimal ratios of acidic/basic sites, Brønsted/Lewis acid sites ratio, as well as the catalyst’s large surface area.

**Figure 15:**
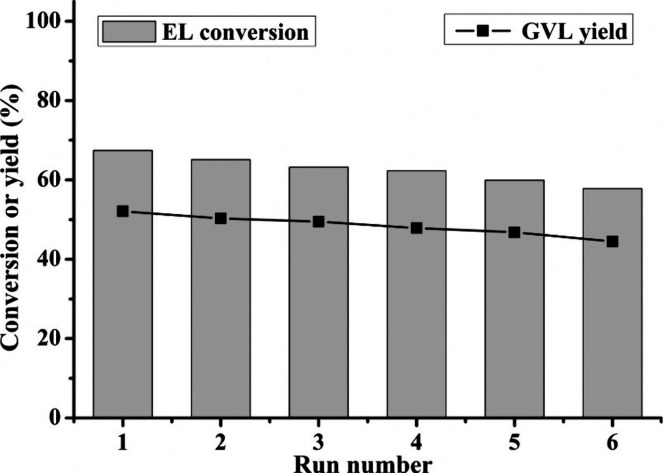
Recyclability of regenerate Ti_2_Zr_8_ in the liquid phase batch mode GVL production from EL. Reproduced with permission from ref (67). Copyright (2017) American Chemical Society.

In a different study, Yang et al. investigated the performance of porous Ti/Zr microspheres in the production of GVL from EL with 2-propanol as a hydrogen donor.^67^ A series of microsphere Ti_*x*_Zr_*y*_ mixed oxide catalysts with different Ti/Zr molar ratios were synthesized via a sol–gel process combined with solvothermal treatment. It was observed that Ti_2_Zr_8_ was the most active, as complete EL conversion was achieved with a 90% GVL yield. This was attributed to the following physiochemical properties: large surface area (385 m^2^ g^–1^), moderate acidity (1.12 mmol g^–1^) and low basicity (0.46 mmol g^–1^). Additionally, as Figure 15 shows, the Ti_2_Zr_8_ catalyst was stable through six consecutive recycles without a significant depreciation in yield.

Similarly, Sakakibara et al. evaluated the productivity of a nickel on zirconium oxide (Ni/ZrO_2_) catalyst in the MPV reduction and lactonisation reaction.^68^ It can be noted that there was a significant uplift in the GVL yield with the addition of Ni/ZrO_2_ compared to pure ZrO_2_. At 120 °C, a 92% yield was obtained, and at 90 °C there was still substantial ML conversion and GVL yield. These findings were credited to the nickel, which contributed to the hydrogenation of the substrate, and the ZrO_2_, which propelled the lactonisation of the hydrogenated product.

**Figure 16:**
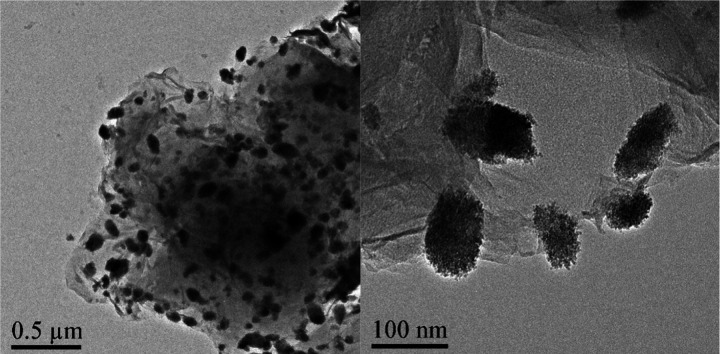
Graphene oxide supported ZrO_2_ (ZrO_2_/GO) catalyst for the transformation of EL to GVL.^69^ Reproduced with permission from Springer Nature.

A novel approach by Lia et al. evaluated graphene oxide (GO)-supported ZrO_2_ (ZrO_2_/GO) catalysts in the transformation of the EL into the desired GVL (Figure 16).^69^ Reaction parameters were moderated to maximize activity and selectivity, and it was reported that 2-propanol worked best as H donor at 180 °C for 10 h. With optimization of these parameters, full EL conversion and 94.8% GVL yield was achieved. When experimentation was conducted exclusively in the presence of GO, no GVL was observed, indicating that the Zr species were the active Lewis acid sites. Moreover, the acidic groups proved to be vital in accelerating the transfer hydrogenation of EL, as they activated the carbonyl group.

Comprehensive studies were conducted by Liu’s group to analyze the acid treatment of the metal-lignocellulosic hybrid, Zr-Pennisetum sinese (Zr@PS).^70^ Lignocellulose derived Pennisetum sinese (PS) is described as containing a mixture of cellulose (40–60 wt %), hemicellulose (15–30 wt %), and lignin (10–25 wt %). PS therefore contains a variety of functional groups such as phenolic hydroxyl groups, alcohol hydroxyl, and carboxyl groups, which assist the ligand coordination with zirconia. Upon the formation of a Zr-based hybrid, the resultant material was modified with organic acids (formic acid, acetic acid, lactic acid, succinic acid, oxalic acid, and citric acid). Notably, formic-acid-assisted Zr@PS (Zr@PS-FA) contributed to a high GVL yield of 96% in 2-propanol at 180 °C for 1.5 h. The Zr@PS-FA was recorded to possess the largest surface area, with enhanced interaction between Lewis and Brønsted acid sites as well as improved catalytic activity from Lewis base sites.

Table 2 summarizes the performance of Zr-based catalysts presented in this section in the CTH of LA and some of its esters to GVL under various reaction conditions. These studies have shown the significance of the acid/base strengths, ratios of Lewis:Brønsted sites, high concentration, and surface areas for an increased catalytic activity and improved conversion and selectivity in the CTH reaction.

**Table 2:** Overview of Zr-Based Catalysts Used in GVL Production Using Alcohols as the H Donor

Catalyst	Reactant	H donor	Catalyst:Reactant/wt %	Reaction temperature/°C	Conversion/%	GVL yield/%	Productivity/mmol_GVL_ g_cat_ ^–1^ h^–1^	Ref
ZrO_2_	EL (10 wt %)	Methanol	9.5	250	25	<1	0	(52)
ZrO_2_	EL (10 wt %)	Ethanol	9.5	250	12	8	0.7	(52)
ZrO_2_	EL (10 wt %)	2-Propanol	9.5	250	31	27	2.5	(52)
ZrO_2_	EL (10 wt %)	2-Propanol	9.5	300	72	56	5.1	(52)
ZrO_2_	ML (10 wt %)	Ethanol	9.5	250	22	6	0.6	(52)
ZrO_2_	ML (10 wt %)	2-Propanol	9.5	250	21	17	1.7	(52)
ZrO_2_	ML (3.2 wt %)	2-Propanol	15.4	150	55	53	8.8	(48)
*m*-ZrO_2_	ML (22 wt %)	Ethanol	Continuous flow	250	>99	63	-	(55)
*t*-ZrO_2_	ML (22 wt %)	Ethanol	Continuous flow	250	>99	66	-	(55)
ZrO_2_ (10 wt %)/SBA-15	ML (3.2 wt %)	2-Propanol	157	150	>99	91	1.5	(48)
ZrO_2_ (11.6 wt %)/SBA-15	EL (4.6 wt %)	2-Propanol	13.9	170	55	40	3.3	(49)
ZrO_2_ (11.6 wt %)/SBA-15	EL (4.6 wt %)	2-Propanol	Continuous flow	150	50	31	-	(49)
ZrO_2_ (23 wt %)/SBA-15	LA (21.6 wt %)	2-Propanol	Continuous flow	250	>99	93	-	(62)
SZ + ZrO_2_ (10 wt %)/SBA-15	LA (1.9 wt %)	2-Propanol	Continuous flow	150	86	56	-	(50)
ZrO_2_ (10 wt %)/KIT-5	EL (3.5 wt %)	2-Propanol	69	180	94	86	2.1	(64)
(ZrO)_2_(OH)_2_CO_3_	EL (3.2 wt %)	2-Propanol	35.6	180	>99	96	6.9	(63)
ZrO_2_/GO	EL (1.8 wt %)	2-Propanol	13.9	180	>99	95	4.7	(69)
Al_2_O_3_–ZrO_2_	EL (3.2 wt %)	2-Propanol	55.4	220	96	83	3.3	(65)
Ni/ZrO_2_	ML (6 wt %)	2-Propanol	100	120	>99	92	0.4	(68)
Ni/ZrO_2_	LA (6 wt %)	2-Propanol	100	120	>99	88	0.4	(68)
Zr-CA	EL (2.35 wt %)	2-Propanol	139	150	>99	97	1.2	(56)
Zr-CA	LA (1.9 wt %)	2-Propanol	172	150	>99	97	2.5	(56)
Zr-GA	EL (3.5 wt %)	2-Propanol	139	160	>99	94	0.6	(57)
Zr-GA	LA (2.9 wt %)	2-Propanol	172	160	>99	97	1.2	(57)
Zr-PO	BL (4.2 wt %)	2-Propanol	29	210	98	96	9.7	(58)
Zr-TMPA	EL (3.5 wt %)	2-Propanol	139	160	>99	96	0.6	(59)
Zr-HEDP	EL (3.5 wt %)	2-Propanol	139	160	99	92	0.4	(60)
Zr-TiO_2_	EL (3.6 wt %)	2-Propanol	23.1	190	79	74	4.4	(66)
GluPC-Zr	LA (21.2 wt %)	2-Propanol	8.6	190	>99	98	8.2	(61)
Zr@PS-FA	LA (1.9 wt %)	2-Propanol	33	180	>99	96	16.5	(70)
Ti_2_Zr_8_	EL (3.2 wt %)	2-Propanol	55.3	180	>99	90	2.2	(67)

#### 2.3.2 Zeolite Catalysts

**Figure 17:**
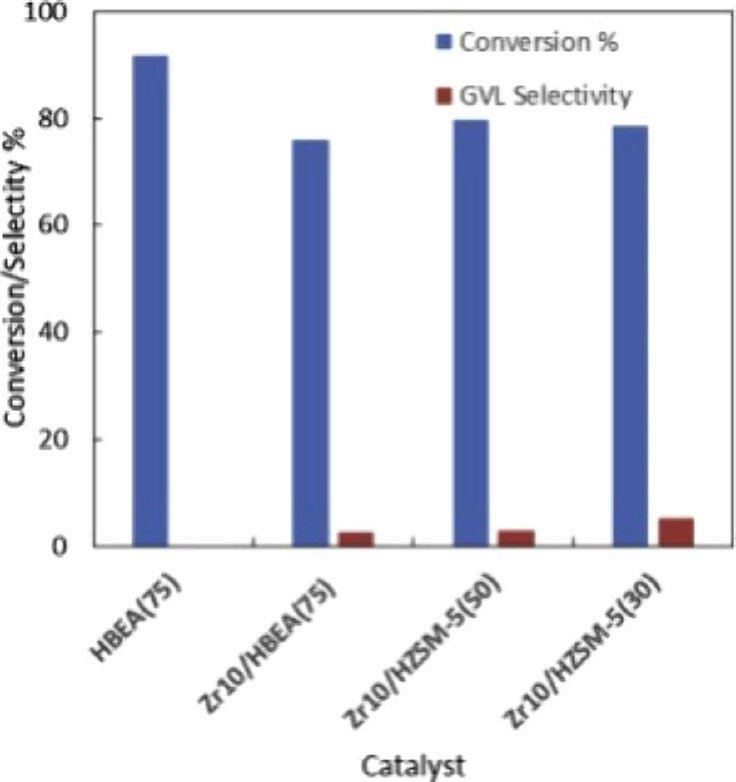
Catalytic activity of HBEA(75) and 10 wt % ZrO_2_ on zeolites in the continuous flow ML transfer hydrogenation. Reaction conditions: liquid phase continuous flow reaction using 0.3 M ML in 2-propanol, flow rate of 0.2 mL min^–1^, 0.5 g of catalyst, 200 °C, and 30 bar pressure. Reproduced from ref (71). Copyright (2019), with permission from Elsevier.

Zeolites are microporous aluminosilicate materials with crystalline frameworks. Their Lewis and Brønsted acidities make them potentially excellent catalysts for MPV reduction, particularly for the transformation of LA and its esters to GVL. A group of researchers led by Pineda used BEA-75 (Si/Al = 37.5) and ZSM-5 (with two different Si/Al = ratios of 15 and 25, respectively) as support for zirconia and tested them in the continuous flow transfer hydrogenation of ML with 2-propanol as the H donor.^71^ Interestingly, they observed considerable conversion (>75%), but almost negligible selectivity to GVL, as shown in Figure 17. They found that the main reaction products were LA produced via hydrolysis of ML, and the subsequent transesterification product isopropyl levulinate, both with selectivity values around 40% and 60%, respectively. They attributed the side reactions to catalysis by the acid sites present on zeolites. Subsequently, they performed poisoning of acid sites with different bases (pyridine, which interacts with both Lewis and Brønsted acid sites, and 2.6-dimethylpyridine, which mainly interacts with Brønsted acid sites) to better understand the role of the catalyst acidity in the CTH reaction. Controlled poisoning of ZrO_2_-doped HBEA(75) and HZSM-5(50) with pyridine led to a decrease in the activity but a considerable improvement in GVL selectivity (>90%). Poisoning with 2.6-dimethylpyridine, which exhibited stronger interaction with Brønsted acid sites, also resulted in a decrease of the activity and drastic increase to complete GVL selectivity. They concluded that Brønsted acid sites favored ML hydrolysis and transesterification reactions that compete with the MPV transfer hydrogenation process of ML. They proposed that the formation of alkoxide species between ZrO_2_ and the hydrogen donor solvent were responsible for the hydrogen transfer.

López-Aguado et al. have reported the chemistry of Zr–Al-beta zeolite catalyst, synthesized via the postsynthetic modification of a dealuminated commercial beta zeolite.^72^ The bifunctional Zr–Al-beta was then catalytically tested using LA and 2-propanol as a hydrogen donor over 20 days in continuous operation at 170 °C. Notably, stable sustained operations resulted in high LA conversion (95%) and GVL yield (90%). The success and reaction productivity were credited to the tailored ratio of Lewis:Brønsted acid sites, which provided catalyzed hydrogen transfer and acid-catalyzed transformations in conjunction.

**Figure 18:**
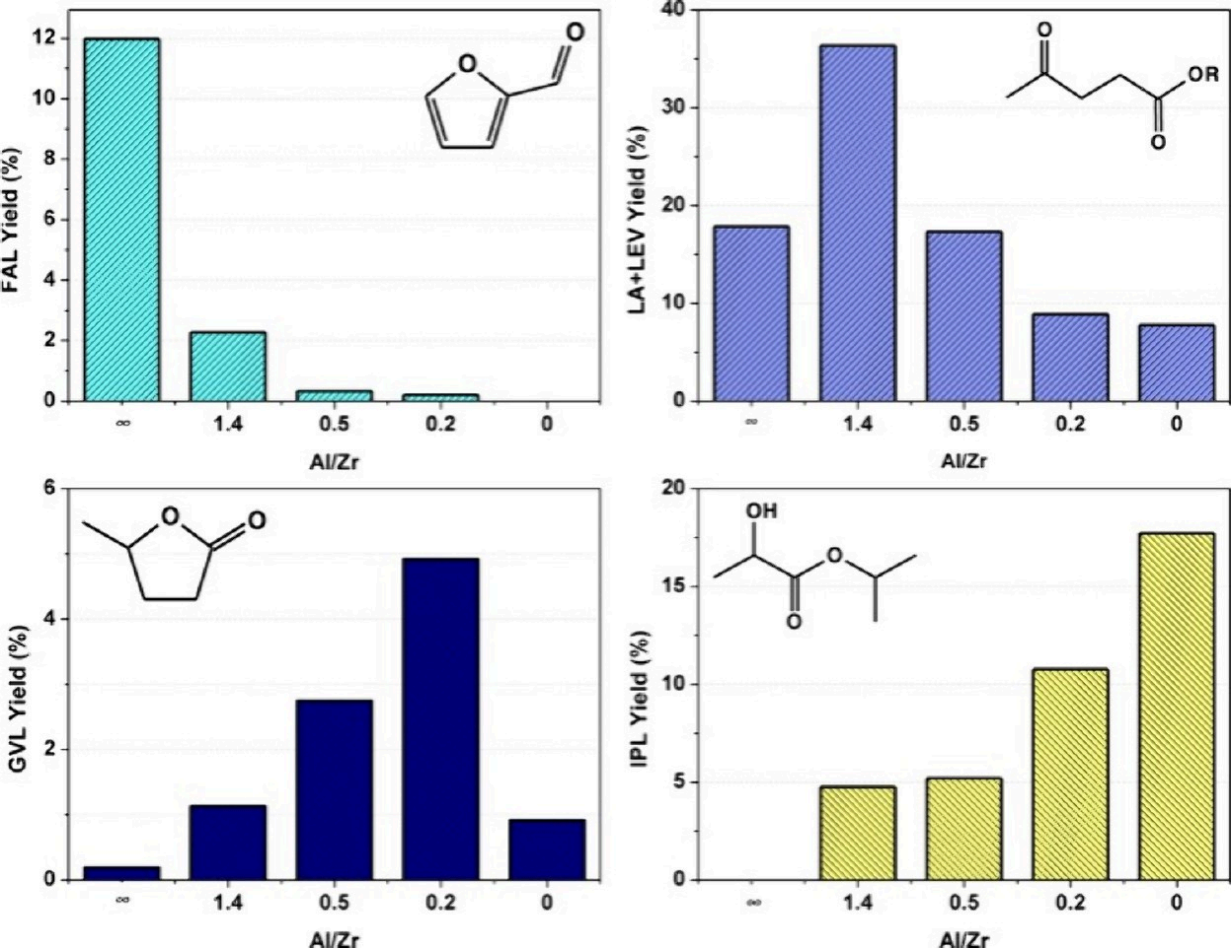
Yields of different products of one-pot transformation of glucose over Zr–Al-Beta zeolites with various Al/Zr molar ratios (FAL = furfural, LA = levulinic acid, LEV = alkyl levulinate, GVL, and IPL = isopropyl levulinate) in the one-pot liquid phase transformation of glucose over commercial Al-Beta zeolite and Zr–Al–Beta modified zeolites. Reaction conditions: temperature: 170 °C; catalyst loading: 15 g L^–1^; 2-propanol:glucose = 40:1 (mol); reaction time: 8 h. Reproduced from ref (73). Copyright (2021), with permission from Elsevier.

The same research group investigated further the performance of Zr–Al-beta zeolite in one-pot transformation of glucose to GVL.^73^ They demonstrated that adjusting the Al and Zr content in the synthesized zeolite determined the preferred reaction, influencing the final product distribution (Figure 18). In the parent Al-Beta zeolite, the absence of Zr sites primarily promoted furfural formation. However, gradually replacing Al with Zr in the zeolitic framework enhanced the formation of GVL. Conversely, the Al-free Zr-Beta zeolite significantly catalyzed the formation of lactates via the retro-aldol condensation pathway.

**Figure 19:**
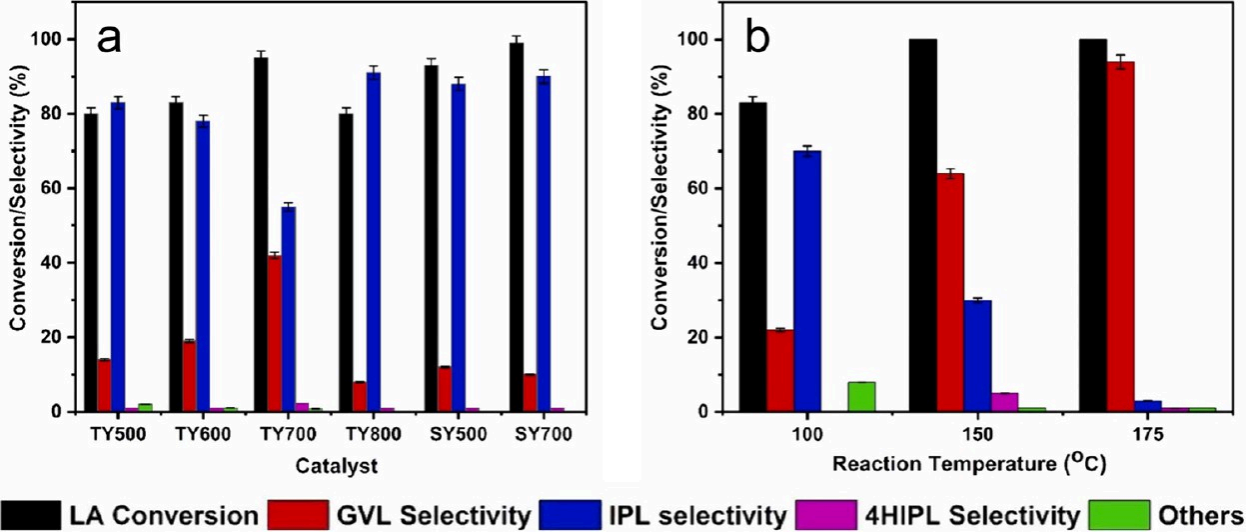
a) Levulinic acid conversion and product selectivity over thermal (TY) and steam (SY) treated Y zeolites at different temperatures and b) effect of reaction temperature on LA conversion over TY700 (reaction conditions: liquid phase reaction in batch mode using 8.6 mmol LA, 500 mg catalyst, 498 mmol 2-propanol, 150 °C, 12 h). Reproduced from ref (74). Copyright (2023), with permission from Elsevier.

Jayakumari and Krishnan prepared various Y zeolites through thermal and steam treatments at different temperatures. The thermal dealumination of NH_4_Y zeolite at 700 °C (TY700) resulted in the highest percentage (21%) of penta-coordinated Al sites. This catalyst showed the highest selectivity (∼94%) for forming GVL under optimized conditions (Figure 19). They proposed that the zeolite’s acid sites catalyze the esterification of LA with 2-propanol, forming isopropyl levulinate, which then undergoes MPV reduction to isopropyl 4-hydroxypentanoate, followed by lactonisation to produce GVL.^74^

Table 3 summarizes the performance of Zr-zeolite-based catalysts for GVL production through CTH reactions.

**Table 3:** Overview of Zeolite Catalysts Used in GVL Production Using Alcohols as H Donors

Catalyst	Reactant	H donor	Catalyst:Reactant	Reaction temperature/°C	Conversion/%	GVL yield/%	Productivity/mmol_GVL_ g_cat_^–1^ h^–1^	Ref
Zr (10 wt %)/HBEA	ML (4.9 wt %)	2-Propanol	Continuous flow	200	80	4	-	(71)
Zr (10 wt %)/HZSM-5	ML (4.9 wt %)	2-Propanol	Continuous flow	200	78	2	-	(71)
Zr–Al–Beta	LA (0.8 wt %)	2-Propanol	Continuous flow	150	68	60	2.2	(72)
Zr–Al–Beta	LA (0.8 wt %)	2-Propanol	Continuous flow	170	95	90	3.4	(72)
Zr–Al–Beta	LA (26 wt %)	2-Propanol	Continuous flow	170	68	42	28.7	(72)
Zr–Al–Beta	LA (26 wt %)	2-Propanol	Continuous flow	190	86	65	44.4	(72)
Zr–Al–Beta	Glucose (6.5 wt %)	2-Pentanol	27.3 wt %	170	90	5	0.13	(73)
Y zeolite	LA (4.1 wt %)	2-Pentanol	100 wt %	175	>99	94	0.67	(74)

#### 2.3.3 Precious Metal Catalysts

While non-noble metal catalysts have shown great efficacy as sustainable alternatives in the CTH of LA and its esters, noble metal catalysts are also particularly effective in both the hydrogenation of unsaturated bonds (e.g., C=O) and the hydrogenolysis of single bonds for bond cleavage (e.g., H–H, C–H, and C–O).^75^

In a method developed by Hsiao et al., the conversion of LA to GVL via CTH was assisted using microwave (MW) heating as opposed to conventional heating (CH), which often involves long reaction times and low yields.^76^ In this study, three precious metal catalysts, including Pd/C, Pt/C and Ru/C, were tested with 2-propanol as the solvent and hydrogen donor. When compared to CH processes, it was concluded that MW assistance significantly improved conversion and resulted in higher GVL yields when using Pt/C and Ru/C catalysts. From this selection of catalysts, Ru/C was the most effective with complete LA conversion and a 99% GVL yield at 160 °C. This study effectively explored the potential of precious metal catalysts in CTH reactions, as well as the promising possibilities of MW as a promising process for enhancing LA conversion and catalytic activity.

**Figure 20:**
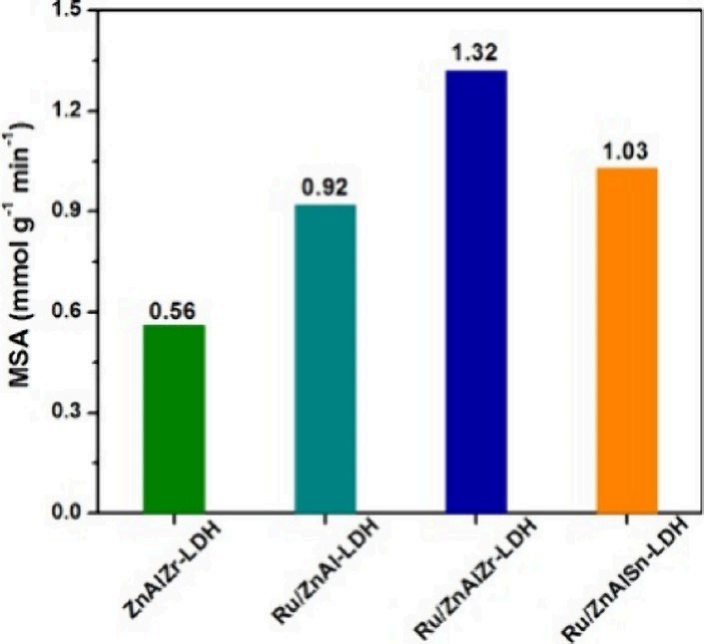
Rate of reaction normalized to the mass of catalyst for various Ru-layer double hydroxides. Reaction conditions: liquid phase reaction in batch mode using 2.8 mmol EL, 131 mmol 2-propanol, 0.1 g of catalyst (Ru loading: 0.9 wt %), N_2_ atmosphere, 200 °C. Reproduced from ref^77^. Copyright (2017), with permission from Elsevier.

**Figure 21:**
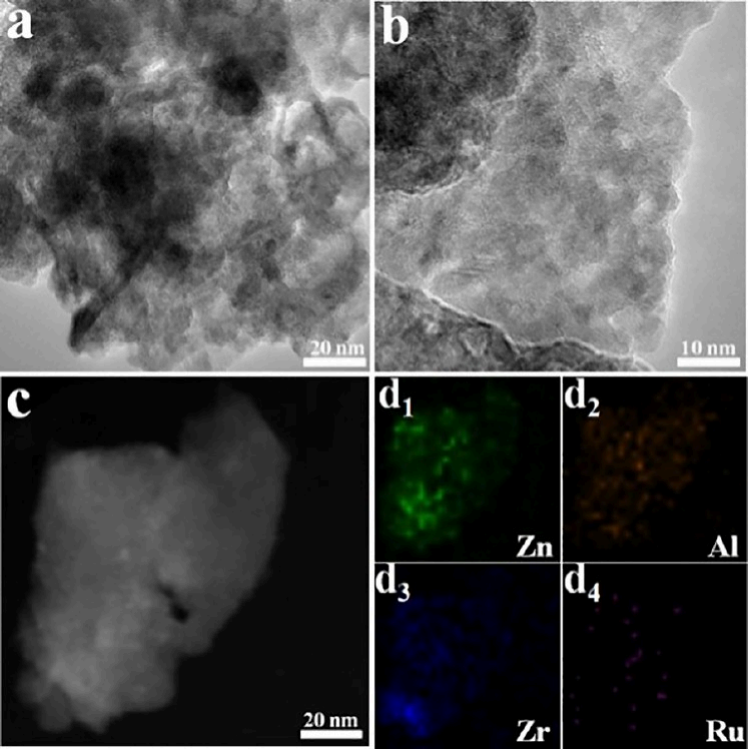
a, b) HRTEM, c) HAADF-STEM image of representative Ru/ZnAlZr-LDH sample, and d) the EDX mapping of Zn, Al, Zr, and Ru. Reproduced from ref^77^. Copyright (2017), with permission from Elsevier.

Three novel ruthenium-based layered double hydroxide catalysts, namely, Ru/ZnAl-LDH, Ru/ZnAlZr-LDH, and Ru/ZnAlSn-LDH, were synthesized by Gao and co-workers.^77^ Comparing the activities of the prepared catalysts, Ru/ZnAlZr-LDH exhibited the highest activity, as illustrated by Figure 20. The catalyst also exhibited outstanding GVL productivity, achieving a remarkable GVL yield of 98% in just 10 min. Moreover, its GVL formation rate of 75 mmol_GVL_ g_cat_^–1^ h^–1^ set a record when compared with other Zr- or Ru-based catalysts that have been previously reported. HRTEM imaging of the Ru/ZnAlZr-LDH sample depicted sheet-like features representative of the LDH moiety (Figure 21a,b). The unprecedented performance of this catalyst was attributed to the cooperative effects of the highly dispersed electron-rich Ru species and the abundance of surface hydroxyl groups on the double-active sites of the ZnAlZr-LDH. HAADF-STEM-EDX images (Figure 21c,d) depicted the uniformity of surface distributions of Zn, Al, Zr, and Ru elements.

The direct conversion of cellulose and GVL has also been a focus of scientific inquiry through integrated alcoholysis and subsequent transfer hydrogenation.^78^ Huang et al. conducted such experiments over mixed metal salt and Ru catalysts on different supports (5 wt % Ru on Al_2_O_3_, active carbon (AC), TiO_2_, and CeO_2_) using 2-propanol as the hydrogen source. Precursory work found that cellulose to levulinates conversion was best catalyzed over an Al_2_(SO_4_)_3_ catalyst. Following this, the subsequent CTH of levulinate to GVL was greatly improved over that of the Ru/ZrO_2_ catalyst. Using microwave heating, at 180 °C and 70 min reaction time, a maximum GVL yield of 51% was reported. Microwave heating also proved to be an effective method for GVL synthesis when compared to conventional oil heating, as it drastically reduced the reaction time. They also tested the 5 wt % Ru/ZrO_2_ in the CTH of ML with microwave heating, achieving complete conversion and 99% GVL yield in just 40 min reaction at 180 °C, proving its effectiveness in CTH of alkyl levulinates. This report attributed the excellent performance of Al_2_(SO_4_)_3_ and Ru/ZrO_2_ mixture catalyst in the one-pot conversion of cellulose to GVL to the synergy between Lewis acidic and basic character of Al_2_(SO_4_)_3_ and the hydrogenation effectiveness of Ru.

It is worth reminding that one of the biggest advantages of CTH over the use of H_2_; gas is the possibility of utilizing inexpensive metal catalysts. Therefore, employing precious metals in CTH is not justifiable unless their performance is significantly superior to that of inexpensive catalysts. This would mean that precious metals should be used in very small quantities, exhibit high stability, offer excellent selectivity, and significantly reduce the process energy requirements. Table 4 provides a summary of the performance of precious metals on different supports for the CTH of LA and some of its esters to GVL under various reaction conditions.

**Table 4:** Overview of Precious Metal Catalysts Used in GVL Production Using Alcohols as H Donor

Catalyst	Reactant	H donor	Catalyst:Reactant	Reaction temperature/°C	Conversion/%	GVL yield/%	Productivity/mmol_GVL_ h^–1^ g_cat_ ^–1^	Ref
Pd (5 wt %)/C	LA (0.7 wt %)	2-Propanol	172 wt %	160	>99	5	0.5	(76)
Pt (5 wt %)/C	LA (0.7 wt %)	2-Propanol	172 wt %	160	95	77	7.7	(76)
Ru (5 wt %)/C	LA (0.7 wt %)	2-Propanol	172 wt %	160	99	82	8.2	(76)
Ru (5 wt %)/C	LA (0.7 wt %)^a^	2-Propanol	172 wt %	160	>99	>99	10	(76)
Ru (1 wt %)/ZnAlZr-LDH	EL (4.9 wt %)	2-Propanol	50 wt %	200	94	98	75	(77)
Ru (5 wt %)/ZrO_2_	ML(3.4 wt %)^a^	2-Propanol	26 wt %	180	>99	99	44.6	(78)
Al_2_(SO_4_)_3_ + Ru (5 wt %)/ZrO_2_	Cellulose^a^	2-Propanol	21 wt %	180	>99	51	13.2	(78)

a Microwave heating.

#### 2.3.4 Non-precious Metal Catalysts

Apart from zirconia, precious metals, and zeolites, some other transition metal catalysts have also been investigated as promising catalysts for the transformation of LA and its esters to GVL via CTH. These metals are often immobilized on a variety of organic or inorganic supports. The fine-tuning of the acidity and basicity of these supports has been shown to influence significantly the activity and productivity during CTH reactions.

For example, Cao et al. prepared a Ni/V_2_O_5_ catalyst by solid phase grinding of nickel citrate complex and V_2_O_5_ and subsequent *in situ* reduction.^79^ From this, experimentation proceeded using EL as reactant and 2-propanol as the H donor solvent at 180 °C for 4 h. A 97% EL conversion and a 92% GVL yield was accomplished using 30% Ni/V_2_O_5_ as the catalyst. The superior activity of this catalyst, compared to other supports tested (Fe_2_O_3_ and Al_2_O_3_), was attributed to the appropriate amount of acidic active sites in Ni/V_2_O_5_, which promoted the lactonization of 4-HPE. Additionally, the catalyst retained its stability for up to five reaction cycles, with minimal depreciation in GVL selectivity.

**Figure 22:**
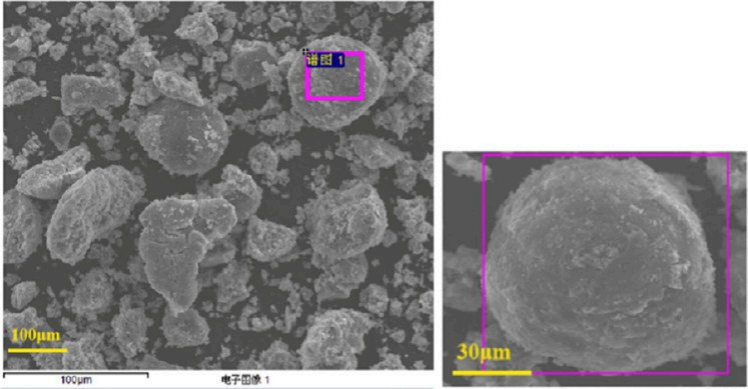
SEM images of equilibrium fluid-catalytic-cracking catalysts (Ni/E-cats).^51^ Reproduced by permission from Springer Nature.

Works by Chen et al. focused on preparation and testing a Ni-based catalyst, supported on equilibrium fluid-catalytic-cracking catalysts (Ni/E-cats), for an EL to GVL transformation, using 2-propanol as H donor solvent.^51^ E-cat acid support is inexpensive and sustainable as it is obtained from waste zeolite catalyst from the fluid-catalytic-cracking process in oil refineries. SEM images of the resultant catalysts can be obtained in Figure 22. Catalyst preparation involved a grinding-pyrolysis method, in which Ni(NO_3_)_2_·6H_2_O precursor and C_6_H_8_O_7_·H_2_O were mixed, ground, and dried to yield the desired Ni/E-cats with different Ni loadings. At 180 °C for 6 h over 30-Ni/E-cat a 90% EL conversion and 87% GVL was obtained. Thorough analytical characterization revealed that the elevated activity of the 30-Ni/E-cat catalyst could be attributed to a high dispersion of Ni metal active centers and significant availability of acidic sites. From further characterization analyses, it was observed that metal and acid sites of Ni/E-cat played a synergistic role in GVL production. Specifically, Ni metal sites were found to assist the hydrogenation of the ketone group in EL and the acid sites of E-cat stimulated the lactonisation of the alkyl 4-hydroxyvalerate intermediates. Additionally, Ni/E-cat catalyst was considerably stable with no obvious loss of catalytic activity for up to four cycles.

**Figure 23:**
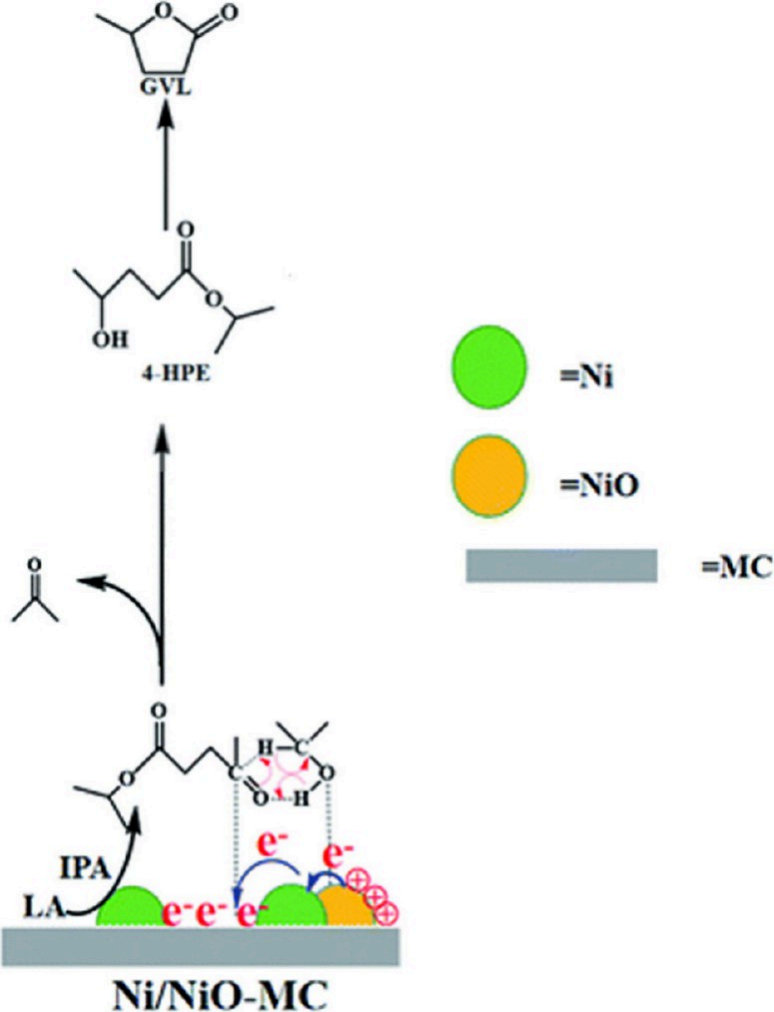
Possible mechanism for the transfer hydrogenation of LA to produce GVL over Ni/NiO–MC. Reproduced from ref^80^ with permission from the Royal Society of Chemistry.

Liu and Li explored Ni and NiO nanoparticles distributed on mesoporous carbon (Ni/NiO–MC) prepared through the pyrolysis of a Ni/mesostructured polymeric gel precursor.^80^ This Ni–polymeric gel precursor was synthesized via ion-exchange between Ni^2+^ in [Ni(NH_3_)_6_]^2+^ and H^+^ in the polymeric gel. The synergistic effect from the Ni, NiO and mesoporous carbon components resulted in superior performance for the transfer hydrogenation of LA providing a complete LA conversion and a 99.5% GVL yield with a turnover frequency of 10.80 h^–1^ at 200 °C in 2-propanol. The combined presence of Lewis acid sites and basic sites on MC promoted the conversion of LA to GVL. Particularly, the Lewis acid sites could facilitate the esterification of LA, while the basic sites acted as the adsorption site for the C=O bond in isopropyl levulinate. The 2-propanol could then be adsorbed on the acid sites, releasing a proton for transfer to the C=O bond via a concerted method involving a six-membered ring transition state to the form 4-hydroxypentanoic acid ester (4-HPE), with successive release of acetone. Following this, the cyclization of the 4-HPE could lead to the formation of GVL (Figure 23). While the catalyst performance is promising, it should be noted that these results were obtained under conditions employing a very dilute solution of the reactant (0.15 wt %).

Following greater interest into Ni-based catalysts, Yu et al. prepared a range of Ni_3_P-CePO_4_ catalysts via a H_2_ temperature-programmed reduction method.^81^ Upon synthesis, these catalysts were utilized in the transfer hydrogenation of LA to GVL using 2-propanol as H donor solvent. Subsequently, a series of experiments were conducted to evaluate the effects of the Ce/Ni molar ratio, reaction temperature, and reaction time. Under optimal conditions at 180 °C for 2 h over the Ni_3_P-CePO_4_(0.1) catalyst a 99% LA conversion and a 90% GVL yield was achieved. NH_3_-TPD and CO_2_-TPD analyses along with poisoning experiments demonstrated synergic roles of acidic and basic sites. The group concluded that the metal–acid/base pairs were vital for the dehydrogenation, C–O cleavage, dehydration, esterification, and (retro-)aldolisation for the formation of GVL. Furthermore, the analysis of the fresh and spent catalysts revealed that there was neither leaching of the active sites, nor changes to the crystalline structure, nor alteration in acidic/basic properties, thus catalyst deactivation was attributed to the deposition of insoluble organics or polymers.

In a notable study, Cia and co-workers discovered that the synergy between Cu and Ni particles in a Cu–Ni bimetallic catalyst were responsible for the high stability and reactivity during the CTH of EL to GVL with 2-butanol.^82^ This group reported that 10Cu-5Ni/Al_2_O_3_ exhibited the highest activity with a 97% yield of GVL in 12 h at 150 °C. Upon comparison with only Cu catalysts, along with a series of poisoning experiments, revealed that the introduction of Ni to Cu considerably boosted the catalyst’s activity and stability. This also resulted in a noteworthy recyclability of 10 runs without a notable loss in the activity. Despite the promising performance in EL conversion, the catalyst exhibited limited activity in LA conversion, only reaching 10% under similar conditions.

Similarly, Yu et al. aimed to synthesize an activated carbon-supported hybrid catalyst for CTH of LA, in which CuNi nanoparticles serve as hydrogenation sites and Al oxide as acid sites.^83^ These were highly dispersed on the surface of active carbon to act as a stable support. The resultant CuNi-1Al/AC catalysts with 5 wt % CuNi alloy and 5 wt % Al proved promising in the production of GVL with full LA conversion and 97% GVL yield at 220 °C in 2-propanol for 2 h. Enhanced activity of the catalyst is credited to the esterification assisted by the acid sites on the AC support. The Al-modification also showed activity for the esterification reaction with various alcohol hydrogen donors. Despite exhibiting excellent activity, the catalyst lost its activity during the course of the reaction due to the shedding of the active components. They suggested boosting the stability of the catalyst with an improved stepwise impregnation of the catalyst.

**Figure 24:**
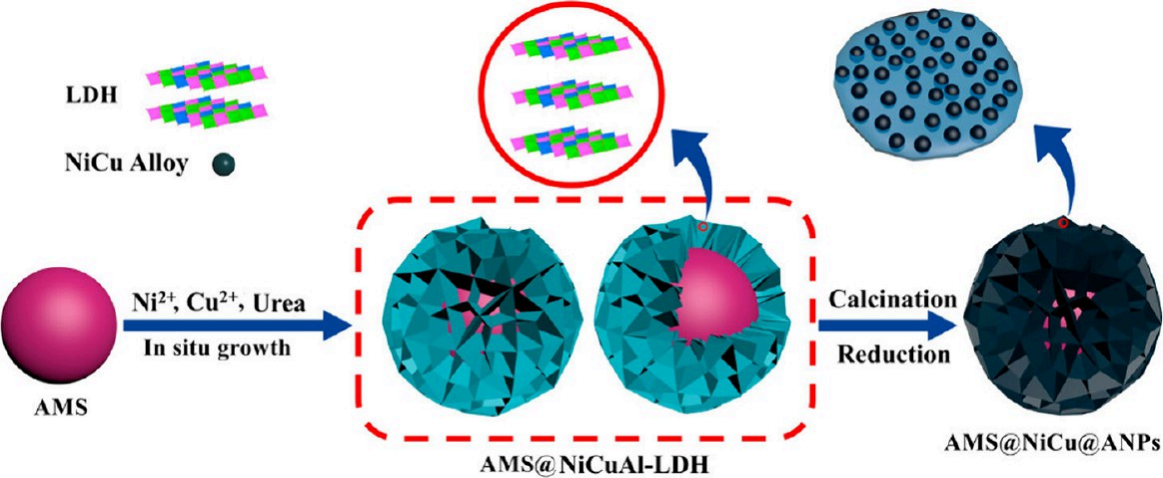
Synthetic procedure for AMS@NiCu@ANPs through transformation of the AMS@NiCuAl-LDH precursor. Reproduced with permission from ref^84^. Copyright (2019) American Chemical Society.

**Figure 25:**
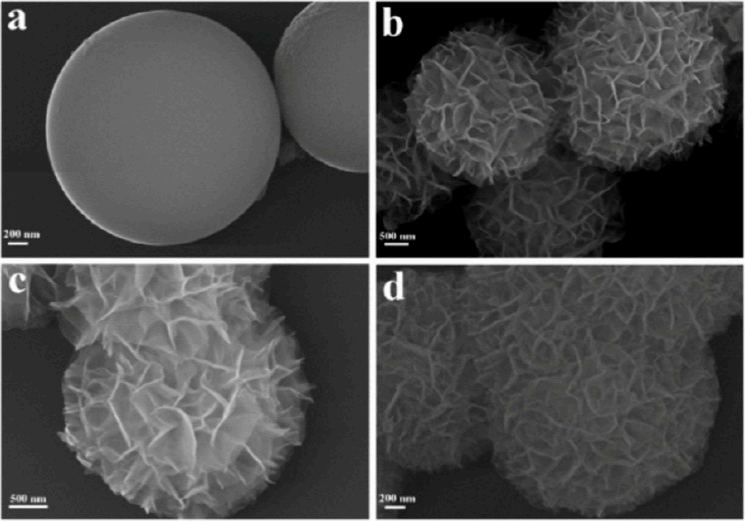
SEM images of a) alumina microsphere (AMS), (b, c) AMS@LDH-0.67, and d) NiCu-0.67. Reproduced with permission from ref^84^. Copyright (2019) American Chemical Society.

Liu et al. also made similar efforts to convert EL to GVL through CTH using a bimetallic NiCu catalyst with an alumina microsphere (AMS) support fashioned into a hierarchical flower-like structure (AMS@NiCu@ANPs).^84^ Synthesis of this novel catalyst developed from a flower-like core–shell structured AMS@Ni–Cu–Al layered double hydroxide precursors (AMS@NiCuAl-LDH), as shown in Figure 24. Scanning electron microscopy (SEM) imaging (Figure 25) representative of AMS@LDH-0.67 and NiCu-0.67 samples confirmed the formation of hierarchical flower-like microstructures after *in situ* direct growth of LDH crystallites on the surface of spherical AMS. It was demonstrated that Cu/Ni ratio had a significant influence on the activity of the catalyst, with a Ni/(Ni + Cu) molar ratio of 0.67 exhibiting the best catalytic performance. This was due to the enhanced surface acid–base properties, relating to the electronic effect. This particular catalyst achieved an EL conversion of 92%, with the highest GVL yield of 82% in 6 h. The group attributed the high catalytic activity on several factors including the synergy between Ni–Cu species in bimetallic NiCu nanoparticles (NPs), which aided the adsorption and activation of EL substrate; the ample acidic and basic sites promoting the MPV reduction process, and the improved porous superstructure. Furthermore, the catalyst retained good stability during reusability cycles of up to five runs which was linked to the strong interactions between the alumina nanoplatelets (ANPs) and the AMS support and between the NiCu NPs and the ANPs matrix.

For the synthesis of an activated carbon-supported copper catalyst (Cu/AC), Gong et al. employed the use of a facile ultrasound-assisted impregnation method with the aid of carbothermal reduction of the AC.^85^ The resultant catalyst was effective for the conversion of both furfural to 2-methylfuran and LA to GVL using 2-propanol as a H donor, within 5 h at 200–220 °C. The high GVL selectivity of 90% and complete LA conversion was attributed to the uniform size and high dispersity of Cu nanoparticles supported on a large surface area AC with an optimal proportion of Cu^2+^, Cu^+^, and Cu^0^ species. It was proposed that the Cu^0^ species acted as active centers for dehydrogenation of 2-propanol to generate active H^+^, while the electrophilic Cu^+^ species captured the LA, facilitating the MPV reaction.

**Figure 26:**
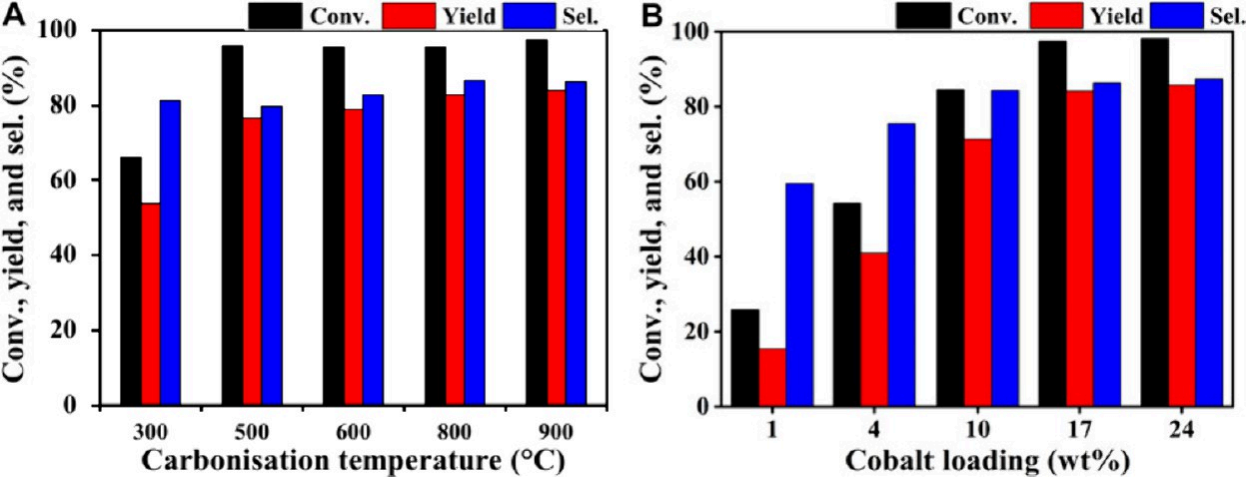
Performance of tannic acid carbon supported cobalt catalysts a) made with different carbonization temperatures and b) with various cobalt loadings for the conversion of EL to GVL. Reaction conditions: Liquid phase reaction in batch mode using 1 mmol of EL, 5 mL of 2-propanol, 100 mg of catalyst, 0.1 MPa N_2_ 150 °C, 3 h. Reproduced with permission under a Creative Commons CC-BY license (CC-BY 4.0) from ref^86^. Copyright (2022) Frontiers Media.

The transition metal cobalt on a tannic acid carbon support (Co/TAC-T) was utilized in the CTH of EL to GVL along with 2-propanol as hydrogen source.^86^ Enquiring into the optimal preparation and reaction conditions found that a carbonization temperature of 900 °C for the support was most effective for the activity of the catalyst (Figure 26a). This is because catalyst Co/TAC-900, compared to supports carbonized at 300, 500, 600, and 800 °C, had more strongly acidic sites and less strongly basic sites, which promoted the transfer hydrogenation of the substrate and reduced side reactions. Furthermore, an increase in cobalt loading significantly improved catalytic activity, with a loading of 17 wt % leading to better conversion (Figure 26b). However, further increasing the cobalt loading to 24 wt % did not result in a significant increase in activity. Under the optimized reaction conditions (150 °C for 3 h), the Co/TAC-900 catalyst attained a 97.5% conversion with a 84.1% GVL yield.

In Xie et al.’s work, an organic–inorganic hybrid catalyst containing hafnium (Hf-ATMP) was prepared using HfCl_4_ and amino tri(methylene phosphonic acid) the CTH of LA using 2-propanol.^87^ Using a 52 mol % Hf-containing catalyst and under optimal conditions of 150 °C and 4 h, a total 95% conversion with 86% GVL selectivity was observed. Furthermore, Hf-ATMP showed no notable decrease in activity and selectivity for up to five cycles. Systematic studies revealed that the porosity of the prepared catalyst and the acidity of Hf and the basicity of the phosphate groups were responsible for the superior catalytic activity observed in the study.

*In lieu* of agricultural waste concerns, Huang et al. aimed to utilize sugar cane bagasse (SB), the main byproduct of cane sugar manufacturing industry, for the synthesis of a Hf-bagasse coordination complex derived catalyst.^88^ Preparation of this catalyst involved a one-step hydrothermal method to assemble SB with HfCl_4_ and methanesulfonic acid (MSA) producing Hf@SB-MSA. Following a range of catalyst characterisations, it was concluded that the coordination between Hf^4+^ and phenolic hydroxyl, alcohol hydroxyl, and carboxyl groups in SB resulted in the formation of Lewis acid and Lewis base sites, while sulfonic group in MSA provided Brønsted acid sites. The collective synergistic effects of Lewis and Brønsted acids and Lewis bases in Hf@SB-MSA enabled 99% GVL yield in the CTH of LA to GVL using 2-propanol as a H donor solvent. Furthermore, recycling experiments revealed the reusability of Hf@SB-MSA upon filtration with at least five reaction runs without an obvious loss of activity.

With aims to develop a simple, efficient, and economical catalyst for the CTH of LA and its esters, Jori and Jadhav synthesized a new Hf-based carbonaceous catalyst (Hf@CCSO_3_H) via simultaneous carbonization and sulfonation of readily available glucose, followed by incorporation of Hf on the surface of the catalyst.^89^ At 150 wt % of catalyst at 200 °C for 24 h in a 2-propanol solvent as a hydrogen donor enabled complete conversion of levulinic acid to be achieved with an excellent 99% conversion and a 96% GVL yield. However, this reaction proceeded over a long period of time with a productivity of 0.2 mmol_GVL_ g_cat_^–1^ h^–1^. X-ray powder diffraction (XRD) analysis revealed that the Hf@CCSO_3_H catalyst was amorphous in nature, which allowed for a higher surface area and thus better activity. The acidic sites on the catalyst assisted the dissociation of 2-propanol into a proton and the corresponding isopropoxide anion, while the carbonyl group in LA was activated by the Hf on the surface of the catalyst.

The conversion of biomass-derived LA into GVL has led to keener interest in the catalytic effects of modified Sn and silica supports. For example, Xu and colleagues synthesized a Sn-modified silica catalyst (SnO_2_/SBA-15) which afforded a high LA conversion of 85% and 81% GVL yield.^90^ The good catalytic performance of SnO_2_/SBA-15 was attributed to the combined Lewis and Brønsted acidity as these sites catalyze the individual steps of hydrogen transfer and esterification for the upgrading of LA to GVL. In a more recent work, Kumaravel et al. developed a family of Sn-loaded Al-SBA-15 catalysts [*x*% Sn/Al-SBA-15 (*x* = Si/Sn = 10, 25, 50, 75, and 100 with Si/Al = 25)] using a hydrothermal *in situ* method.^91^ Under mild reaction conditions (200 °C, 2-propanol, 3 h) Sn/Al-SBA-15 (Si/Sn = 25) demonstrated 99%GVL yield. The characterization experiments revealed high dispersity of Sn species in the uniform pore channels of Al-SBA-15, which contributed to the good performance of the catalyst.

**Figure 27:**
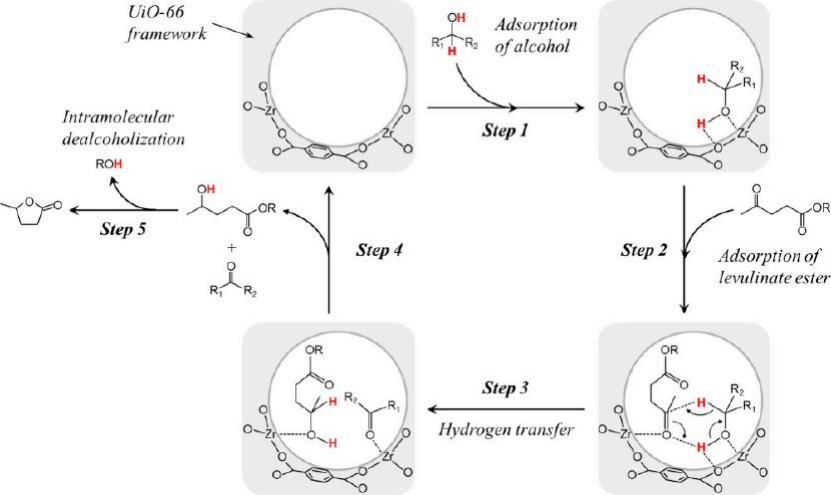
Possible reaction mechanism for CTH of levulinate esters to produce GVL over sulfonic acid-functionalized UiO-66. Reproduced with permission from ref^92^. Copyright (2017) American Chemical Society.

Kuwahara et al. prepared a sulfonic acid functionalized UiO-66, which is a type of metal–organic framework.^92^ They employed this catalyst in ML conversion to GVL using 2-butanol as a hydrogen donor and achieved 80% GVL yield at 140 °C after 9 h. Under similar reaction conditions but using LA as the reactant, a 69% conversion and 25% GVL yield was obtained. By performing comparative studies, they found that the strong catalytic performance came from symbiotic effects between Lewis-basic Zr_6_O_4_(OH)_4_ clusters and Brønsted-acidic −SO_3_H sites. These were situated closely together in a restricted nanospace and worked together to catalyze the reaction of CTH for LA and its esters, helping with the consecutive intramolecular dealcoholization that produces GVL (Figure 27). The catalysts exhibited good stability over 4 cycles of reaction.

Cao et al. prepared a series of Cu–Mg oxides with varying Cu/Mg molar ratios using a modified urea-precipitation method.^93^ They proposed a two-step strategy for GVL production, integrating ML formation through H_2_SO_4_-catalyzed methanolysis of cellulose to form an ML-rich solution, followed by CTH of ML to GVL. In their experiments, they found that after a 1 h reaction at 220 °C between ML and methanol, CuO achieved a 62% conversion with 89% selectivity to GVL. In comparison, MgO showed only a 2% conversion with 50% selectivity to GVL. A physical mixture of CuO and MgO resulted in a 37% conversion with 86% GVL selectivity. However, the Cu_0.87_Mg_0.13_O_*x*_ catalyst demonstrated a 71% conversion with 90% GVL selectivity, indicating a synergistic effect in the Cu–Mg mixed oxide. By optimizing the reaction conditions, they achieved a 96.4% conversion and 94% selectivity to GVL after 4 h reaction at 220 °C. They identified Cu^+^ species were responsible for the reduction of ML and suggested that the incorporation of MgO led to the coexistence of zero, mono-, and divalent Cu species in Cu_0.87_Mg_0.13_O_*x*_. The strong interaction between MgO and Cu species was found to be the main reason for inhibiting the reduction of the CuO phase in Cu_0.87_Mg_0.13_O_*x*_ during the reaction. This interaction significantly stabilized Cu^+^ species against reduction in a hydrogen atmosphere, greatly facilitating the hydrogenation of ML to GVL.

**Figure 28:**
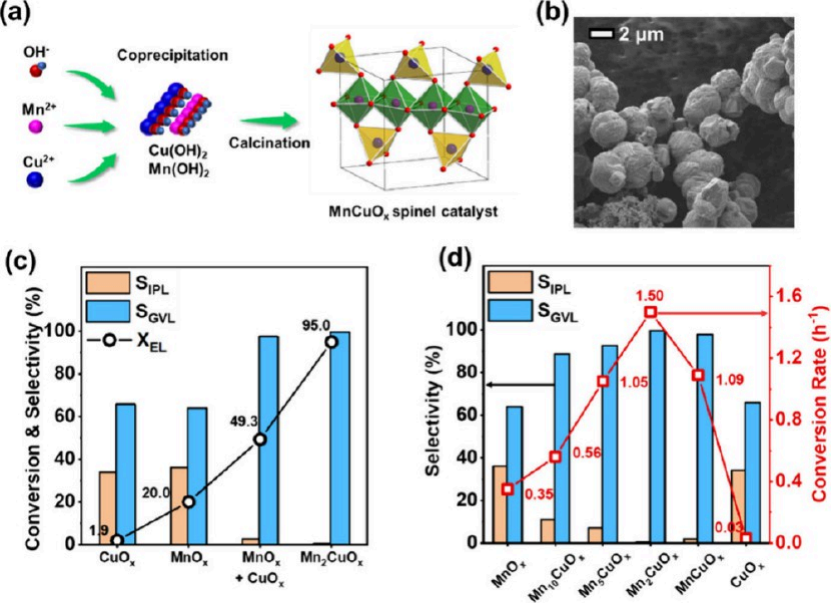
Preparation and synergistic performances of the proposed MnCu oxide catalysts. a) Preparation process of the MnCu oxide catalyst. b) SEM image of the fresh MnCu oxide catalyst. c and d) Catalytic performance of CuOx, MnOx, mixed CuOx, and MnOx and different-atomic ratio bimetallic MnCuOx oxide catalysts, respectively. Reaction condition: Liquid phase reaction in batch mode using 3 mmol of EL, 15 mL of 2-propanol, 0.05 g of catalyst, N_2_ pressure: 1.0 MPa, 200 °C, 3 h, 1000 rpm. Reproduced with permission from ref^94^. Copyright (2022) American Chemical Society.

Xiao Yu and co-workers prepared various bimetallic mixed oxides including CoZnO_*x*_, NiZnO_*x*_, MnCoO_*x*_, CuFeO_*x*_ and MnCuO_*x*_ with 1:1 atomic ratio between the two metals, and tested them in EL CTH.^94^ Among the various bimetallic oxide catalysts investigated, CuFeO_*x*_ and MnCuO_*x*_ catalysts exhibited significantly higher conversions compared to the other three candidates. Particularly, the highest conversion (95%) and GVL selectivity (98%) was achieved using Mn_2_CuO_*x*_ after 3 h of reaction at 200 °C. Comparing the catalytic performance of Mn_2_CuO_*x*_ (Mn/Cu atomic ratio = 2) with the corresponding monometallic oxides and their physical mixtures, as shown in Figure 28c, revealed a strong synergism between Cu and Mn species of the mixed oxide. The monometallic oxides and their admixture were mainly catalyzing the transesterification of EL to isopropyl levulinate at the expense of GVL. They attributed the outstanding performance of the mixed oxide to the formation of unique bimetallic MnCu oxide sites that restrained the transesterification pathway. To better understand the role of each metal oxide and optimize the catalyst formulation, they prepared a series of Mn–Cu mixed oxide catalysts with atomic ratios ranging from 1:1 to 10:1. As presented in Figure 28d, while all bimetallic oxide catalysts show considerably higher selectivity toward GVL (80–97%), Mn_2_CuO_*x*_ displayed the highest selectivity and reaction rate. The concluded that the expanded Mn–O lattice was critical for enhanced transfer hydrogenation of EL to GVL using 2-propanol as the H donor.

The summary of the performance of other transition metal catalysts for the CTH of LA and some of its esters to GVL under various reaction conditions is presented in Table 5.

**Table 5:** Overview of Precious Other Transition Metal Catalysts Used in GVL Production Using Alcohols as H Donor

Catalyst	Reactant	H donor	Catalyst:Reactant	Reaction temperature/°C	Conversion/%	GVL yield/%	Productivity/mmol_GVL_ g_cat_^–1^ h^–1^	Ref
Ni/V_2_O_5_	EL (4.6 wt %)	2-Propanol	30 wt %	180	97	92	5.3	(79)
Ni/Fe_2_O_3_	EL (4.6 wt %)	2-Propanol	30 wt %	180	81	72	4.2	(79)
Ni/Al_2_O_3_	EL (4.6 wt %)	2-Propanol	30 wt %	180	30	26	1.5	(79)
Ni/E-cats	EL (4.6 wt %)	2-Propanol	30 wt %	180	90	87	3.3	(51)
Ni/NiO–MC	LA (0.15 wt %)	2-Propanol	-	200	>99	>99	-	(80)
Ni_3_P-CePO_4_	LA (7.2 wt %)	2-Propanol	8.6 wt %	180	>99	90	45	(81)
10Cu-5Ni/Al_2_O_3_	EL (5.6 wt %)	2-butanol	69 wt %	150	>99	97	0.8	(82)
10Cu-5Ni/Al_2_O_3_	LA (4.6 wt %)	2-butanol	69 wt %	150	10	6	0.05	(82)
CuNi-1Al/AC	LA (0.25 wt %)	2-Propanol	-	220	>99	97	-	(83)
AMS@NiCu@ANP	EL (7.2 wt %)	2-Propanol	16.5 wt %	220	92	82	5.7	(84)
Cu/AC	LA (0.6 wt %)	2-Propanol	100 wt %	220	>99	90	1.5	(85)
Co/TAC-900	EL (3.5 wt %)	2-Propanol	69.4 wt %	150	98	84	2.8	(86)
Hf-ATMP	EL (4.4 wt %)	2-Propanol	139 wt %	150	95	86	1.1	(87)
Hf@SB-MSA	LA (1.3 wt %)	2-Propanol	50 wt %	180	>99	99	1.4	(88)
Hf@CCSO_3_H	LA (3.1 wt %)	2-Propanol	150 wt %	200	>99	96	0.2	(89)
SnO_2_/SBA-15	LA (10.4 wt %)	2-Propanol	40.2 wt %	110	85	81	2.2	(90)
Sn/Al-SBA-15	LA (32.8 wt %)	2-Propanol	-	200	>99	>99	-	(91)
UiO-66-S_60_	ML (3.1 wt %)	2-Butanol	77 wt %	140	98	80	0.9	(92)
UiO-66-S_60_	LA (2.8 wt %)	2-Butanol	86 wt %	140	69	25	0.3	(92)
CuO	ML (2.6 wt %)	Methanol	38.4 wt %	220	62	55	11.0	(93)
MgO	ML (2.6 wt %)	Methanol	38.4 wt %	220	2	1.0	0.2	(93)
CuO+MgO	ML (2.6 wt %)	Methanol	38.4 wt %	220	37	32	6.4	(93)
Cu_0.87_Mg_0.13_O_*x*_	ML (2.6 wt %)	Methanol	38.4 wt %	220	71	69	13.8	(93)
CuFeO_*x*_	EL (3.5 wt %)	2-Propanol	11.6 wt %	200	53	50	10.0	(94)
MnCuO_*x*_	EL (3.5 wt %)	2-Propanol	11.6 wt %	200	69	68	13.5	(94)
Mn_2_CuO_*x*_	EL (3.5 wt %)	2-Propanol	11.6 wt %	200	95	92	18.4	(94)

## 3 Concluding Remarks and Future Directions

GVL is important because it has a wide range of applications and can be derived from renewable energy sources. It holds promise as a fuel additive, precursor to renewable polymers that can replace fossil-fuel-based counterparts, and it is finding its place as a green solvent, especially in biomass processing and related processes. GVL production from biomass is a multistep process, with the hydrogenation step as the key bottleneck controlling the overall yield. Conventional hydrogenation requires expensive metal catalysts and high H_2_ pressure, which are not desirable. This has derived extensive research toward exploring catalytic transfer hydrogenation of levulinic acid and its esters to GVL, which unlike hydrogenation with H_2_ gas, can be performed using inexpensive catalysts in conjunction with liquid hydrogen donating solvents.

In this Perspective, we systematically revised the most recent developments in the area of heterogeneous catalytic transfer hydrogenation of levulinic acid and its esters to GVL. There are different options for the type of catalyst that can be used in this process. It can be observed that the acidity of the catalyst plays a key role here. The type of acid sites is also important. While Lewis acids are required for the hydrogen transfer, Brønsted acids can facilitate the cyclization of the intermediate product. While the effects of the type of acid sites are evident, there is not much insight available in the literature regarding the impact of acid site strength. Basicity is also known to catalyze the reaction, but often it does so into undesirable products. Metal particles can also boost the transformation of LA and its esters into GVL. Ru is a good example of these metals; however, using Ru compromises the economics of the process as it is a precious metal. Non-noble metals such as Ni- and Cu-based catalysts have also shown promise. However, they seem to be sensitive to the presence of air/oxygen, requiring the reaction to be conducted under an inert environment. Several catalysts that have been developed for this process are based on ZrO_2_. However, ZrO_2_ is inherently a low surface area material, and also its selectivity toward GVL leaves room for improvement. Therefore, developing a zirconia catalyst with improved physical properties such as enhanced surface area and improved selectivity would be highly desirable.

More generally, despite all of the progress, several challenges remain. Below, we discuss some of the key challenges that addressing them will speed up the commercialization of sustainable GVL production.

### 3.1 Designing More Efficient and Stable Catalysts

Many researchers have developed catalytic systems that initially appear very promising, reporting high conversion and GVL yields and often performing operating condition optimizations. However, even optimized systems sometimes require long reaction times, high temperatures, or very high catalyst/reactant ratios. This indicates that there is still room for improvement in the design of more efficient catalysts. Catalyst stability is a critical factor in the development of practical and commercially viable catalytic processes. While the demonstrated stability of catalysts over a few cycles in batch processes is promising, scaling up production requires more rigorous testing. Continuous flow reactors offer a more representative simulation of industrial conditions compared with batch reactors, where reactants continuously flow through the catalyst bed. Testing catalysts in continuous flow reactors provides insights into their long-term performance under sustained operating conditions. Stability tests lasting tens of hours or longer are essential to evaluate the catalyst’s durability and activity over extended periods.

### 3.2 Scalability of Catalyst Synthesis

The complexity of catalyst synthesis hinders scalability and commercialization. Many high-performance catalysts require sophisticated and multistep preparation methods. Others may require the use of expensive precursor materials (e.g., in the synthesis of templated materials) or the incorporation of multiple active components, such as bimetallic or trimetallic systems, which add further complexity to the synthesis process. Thus, efficient catalysts with simple and inexpensive preparation methods are highly desirable. Besides the cost, the use of templating agents and hazardous/organic reagents also has implications for the sustainability and Health, Safety and Environment (HSE) credentials of the process. For instance, these agents may be released into effluents or burned off during calcination when operated at a scale. Therefore, efficient catalysts with simple, inexpensive, and environmentally friendly preparation methods are highly desirable.

### 3.3 Understanding the Reaction Mechanism

Gaining insights into the reaction mechanism at the molecular and atomic scale and the role of different catalyst properties is crucial. As highlighted in this article, the number of studies in the literature that have utilized computational methods to provide deeper insights into the interactions of molecules and catalysts at these scales is very limited. Computational tools such as DFT can greatly enhance our understanding of the reaction mechanisms, help identify the nature of the active sites, and elucidate mechanisms of catalyst deactivation. Especially when combined with experimental work, these computational methods can be highly informative, leading to the development of the next generation of efficient and stable catalytic systems.

### 3.4 Engineering Challenges and Scale Up

There are major engineering challenges in the transition from the laboratory-scale synthesis of GVL to industrial production, especially with reactor design. While continuous flow reactors offer scalability and consistency in production, their operation is complicated by heat and mass transfer control problems. These problems become considerably more noticeable when dealing with exothermic reactions, such as hydrogenation. In addition, designing reactors capable of handling a continuous feed of corrosive levulinic acid and the subsequent separation of GVL from the reaction mixture under industrial conditions are other challenges. Another significant challenge is the coproduction of ketones or aldehydes (e.g., acetone or acetaldehyde) and ethers (via etherification) from the sacrificial alcohols used as hydrogen donors in the CTH process. This not only leads to an intrinsically lower atom economy but also necessitates the handling and separation of these byproducts, which can complicate downstream processing. Some of these byproducts may contribute to side reactions that deactivate catalysts and reduce the efficiency of the overall process. Moreover, a greater molar excess of the alcohol donor may be required to drive the reaction toward higher yields of GVL, but this also results in larger volumes of alcohol to be recycled, further impacting the process efficiency and increasing the complexity of solvent recovery systems.

Aside from this, fouling in reactors, catalyst deactivation, and accurate control of the reaction parameters (e.g., temperature, pressure, and flow rates) are important. Lastly, integrating these into present biorefineries and financial feasibility remain major aspects that should be taken into account when implementing them on a larger scale.

Future directions could focus on making the process greener, for instance, by utilizing renewable biomass-derived alcohols. As evidenced by the reviewed literature, secondary alcohols, particularly 2-propanol, are the most commonly used hydrogen donors for the synthesis of GVL via the CTH route, thanks to their higher reactivity compared to primary alcohols. However, this increased reactivity comes at the cost of a higher market price, which negatively impacts the economic viability of the process. Therefore, designing catalysts that allow the use of primary alcohols as hydrogen donors without significantly increasing the energy requirements of the process would be highly beneficial. Ethanol, especially bioethanol, offers significant advantages for the sustainable development of this process, as it is a renewable and widely available resource that aligns well with the principles of green chemistry and sustainability. Additionally, developing multifunctional catalysts that reduce the number of steps in the biomass-to-GVL process would be desirable.
